# Supplementary material for: Unsafe riding practice among electric bikers in Suzhou, China: an observational study
Source: BMJ Open. 2014 Jan 15;4(1):e003902. doi: 10.1136/bmjopen-2013-003902 (PMC3902425; doi:10.1136/bmjopen-2013-003902)
Supplement: Author's manuscript [file bmjopen-2013-003902.draft_revisions.pdf]

Unsafe riding practice among electric bikers in Suzhou: an observational study

Journal:	*BMJ Open*
Manuscript ID:	bmjopen-2013-003902
Article Type:	Research
Date Submitted by the Author:	28-Aug-2013
Complete List of Authors:	Yang, Jie; Jiangsu provincial centre for disease prevention and control, NCD prevention<br>Hu, Yihe; Suzhou municipal centre for disease prevention and control, NCD prevention<br>Du, Wei; Peking University, Institute of Population Research; University of New South Wales, Neuroscience Research Australia<br>Powis, Brent; International Medical University, School of Postgraduate Studies and Research<br>Ozanne-Smith, Joan; Monash University, Department of Forensic Medicine<br>Liao, Yilan; Chinese Academy of Sciences, Institute of Geographical Sciences and Nature Resources Research<br>Li, Ning; Peking University, Institute of Population Research<br>Wu, Ming; Peking University, Institute of Population Research
**Primary Subject Heading**:	Epidemiology
Secondary Subject Heading:	Public health
Keywords:	Electric Bike, Cross Sectional Study, Behavior, Risk Factor Research, Driver

SCHOLARONE™
Manuscripts

For peer review only - http://bmjopen.bmj.com/site/about/guidelines.xhtml

Title

Unsafe riding practice among electric bikers in Suzhou: an observational study

Corresponding Author

Dr. Ming Wu

Tel/Fax: +86 (25) 8375 9411

Email: wuming@jscdc.cn

Address: Department of Non-communicated Chronic Disease Prevention and Control, Jiangsu Provincial Center for Disease Prevention and Control, Nanjing 210009, China

Authors

Jie Yang, MSc[1*], Yihe Hu, MBBS[2*], Wei Du, PhD[3,4], Brent Powis, PhD[5], Joan Ozanne-Smith, MD[6], Yilan Liao, PhD[7], Ning Li, PhD[3], Ming Wu, PhD[1]

1. Department of Chronic Disease Control, Jiangsu Provincial Centre for Disease Control and Prevention, China
2. Department of Chronic Disease Control, Suzhou Municipal Centre for Disease Control and Prevention, Suzhou, China
3. Institute of Population Research, Peking University, China
4. Neuroscience Research Australia, University of New South Wales, Australia
5. School of Postgraduate Studies and Research, International Medical University, Malaysia
6. Department of Forensic Medicine, Monash University, Australia
7. Institute of Geographical Sciences and Nature Resources Research, Chinese Academy of Sciences, China

* These two authors contributed equally

Key Words: Electric Bike, Cross Sectional Study, Behavior, Risk Factor Research, Driver.

Word count: 2,351words

For peer review only

Abstract

Background: Increasing Electric bike (E-bike) related fatalities and injuries may be partly attributable to unsafe riding practice

Objectives: To describe potentially unsafe riding behaviors among E-bikers and to investigate factors influencing these practices in China

Methods: In September 2012, a cross-sectional observation study including a speed measurement component was conducted in Wuzhong (an urban District) and Zhangjiagang (a rural District) of Suzhou, Jiangsu Province, China. Hand held radar speed meters were used to read traveling speeds of E-bikes and a pro-forma observation checklist was used to collect data on road riding practice. Mixed-effect logistic regressions were used to calculate adjusted Odds Ratios (OR) and 95% Confidence Intervals (CI) for speeding, road rule violations, and helmet use.

Results: Among 800 E-bikes with a speed reading, 70.9% exceeded the designed speed limit of 20 km/h. Among a further 20,647 E-bikers observed, 38.3% did not comply with the road rules when entering intersections; and only 2.2% wore helmets. No regional variation was identified between Wuzhong and Zhangjiagang. Male gender was associated with more speeding and road rule violations; whereas riding a pedal-equipped E-bike was associated with less road rule violations and less helmet use.

Conclusion: Unsafe riding practices such as speeding, road rule violations, and lack of helmet use were commonplace among E-bikers, especially males. The study findings suggest that public awareness, road rule revisions and enforcement are needed to discourage unsafe practices and encourage helmet use in order to improve E-bike safety in China.

For peer review only

For peer review only - http://bmjopen.bmj.com/site/about/guidelines.xhtml

Article Summary

Article Focus:

- E-biker safety is an emerging public health challenge in China. This study focuses on the unsafe on-road riding behaviours among Chinese E-bikers and factors influencing these practices.

Key Message:

- Direct roadside observation techniques were applied to describe safety practices of E-bikers and hand held radar meters were used to estimate their actual traveling speed.
- The variation of on-road riding behaviours between rural and urban areas was evaluated.
- Factors influencing observed riding behaviours were further investigated.

Strengths and Limitations of the Study:

- In this study, we evaluated how fast E-bikers ride on roads and the possible regional variation of riding behaviours. Furthermore, we investigated factors influencing observed riding behaviours.
- Study findings could provide new evidence to enhance the understanding of on-road riding behaviours among Chinese E-bikers, and to reinforce the imperative to encourage safety gear use and discourage unsafe on-road practices.
- Findings might be limited by lack of generalizability to other settings, possible bias due to unmeasurable confounding, and possible misclassification due to measurement errors.

For peer review only

INTRODUCTION

In China, the last few years have witnessed the rapid growth of E-bikes (either with pedals or in scooter form) due to increasing mobility demand when public transportation systems are crowded and inconveniently routed.[1] Growing wealth among Chinese also increases affordability of E-bike purchase, normally priced at approximately USD 300. In China, more than 120 million E-bikes were registered by 2011 [2] and globally an estimated 466 million E-bikes are expected to hit the road by 2016. [3]

In China, bicycle use is shifting to E-bike use. Unfortunately, an associated unwanted shift was observed as E-bike related fatalities increased over time from 589 in 2004 to 4,029 in 2010 across the nation, whereas bicycle related fatalities decreased from 13,655 to 4616 during the same period. [4] Moreover, E-bikers hospitalized for injuries accounted for 57% of serious non-fatal road traffic injuries and 50% of the direct hospitalization cost for all road crash casualties in a rural hospital in Suzhou.[5] Thus, E-biker safety is an emerging public health challenge in China .[5-7]

Whilst unsafe riding practices have been reported among E-bikers using a self-reported survey,[2,8] synchronized video camera recording techniques,[9] and direct roadside observations,[10] no studies have reported on how fast E-bikers ride on roads allowing for possible regional variation, such as rural/urban disparities, commonly observed for other road safety issues. To enhance the understanding of on-road riding behaviors among Chinese E-bikers including evaluation of rural/urban variation, we used direct roadside observation techniques to describe their safety practices and hand held radar meters to estimate their actual traveling speed. We

For peer review only - http://bmjopen.bmj.com/site/about/guidelines.xhtml

For peer review only

further investigated factors influencing these observed behaviors.

METHODS

This study comprised two components, i.e., observations *with* or *without* speed measurement, which were conducted separately in Suzhou, one of the intervention pilot cities in China for the Bloomberg Philanthropies Global Road Safety Programme. [11] Suzhou has the sixth highest gross domestic product (GDP) per capita on the Chinese mainland, a resident population of 10 million and at least 2 million E-bikes. [10] The study protocol was approved by the Ethics Committee of Jiangsu Provincial Centre for Disease Control and Prevention.

Field implementation

In Suzhou, two administrative districts, i.e., Wuzhong (urban district) and Zhangjiagang (rural district) were selected to conduct both study components. To select observation sites, a grid was placed over standard maps of Wuzhong and Zhangjiagang, random digits were generated for each grid box for selection and each valid grid box contained at least one intersection having traffic lights. For each randomly selected site, an alternate site was also selected randomly from the grid as a backup. A pilot study was carried out to validate the field feasibility such as having low volumes of E-bikes for speed measurement; at least two-way motor vehicle lanes, pedestrian crossings, and bicycle lanes; enough distance between observation sites so the same E-bikers were unlikely to be observed twice; less likely to interrupt observed behaviors and least likely to increase the crash risk for observers. A total of eight sites (i.e, two from each district for each study component) were randomly selected.

For peer review only - http://bmjopen.bmj.com/site/about/guidelines.xhtml

For the speed measurement component, observers concealed themselves at approximately 50 meters from the corner of the intersection and used handheld radar speed meters (Bushnell Velocity 10-1911CM with measurement range of 16-320km/h) to record the speed meter reading; whereas for the study component without speed measurement, the observations were conducted at intersection corners. We randomly selected 4 days in a week including one weekend day (September 11th, 12th, 13th, and 15th) for the speed measurement component and collected information on on-road riding behaviors among E-bikers during another 7-day period (September 17th-23th). The time of day (7.00am–6.59pm) for site observations was set at 2-hour intervals as observational periods and randomly assigned to sites.

Four experienced observers were recruited, who had participated in previous roadside E-bike observation studies in other districts in Suzhou.[10] Prior to field implementation, the site observers were trained in specifications of different behaviors, identification of different types of protective items, techniques to observe multiple behaviors especially when an E-bike was moving, and data quality control. Roadside pilot observations and regular on-site audits were conducted to ensure the safety guidelines and accurate and appropriate implementation of the data collection process.

The observers worked in pairs, to observe oncoming E-bikes in ascending distance order. Data items were collected on a pro-forma checklist including type of E-bike (with bike pedals or in scooter form), registration status, rider's gender, occupation (courier or not), carrying passengers, carrying oversized cargo (estimated >60 x 40 x 20cm^3, the size of normal airlines carry-on luggage), riding in a motor vehicle lane,

For peer review only - http://bmjopen.bmj.com/site/about/guidelines.xhtml

running red lights, riding in the opposite direction (i.e., facing oncoming traffic), using mobile phone, using helmet, wearing leather gloves, and wearing other motorcycle protective clothing. Weather, day of week, time of day, average E-bike traffic volume per minute, presence of a traffic controller (traffic policeman or traffic police assistant directing vehicular and pedestrian traffic) were recorded on separate data collection forms.[Table 1] Regarding the speed measurement component, for every tenth E-bike, the second observer recorded the radar speed reading during the speed measurement observations.

Table 1. Observational item categorization

Observational items	Categorization
weather	sunny, cloudy, or rainy
day of week	weekday or weekend
time of day	morning or afternoon
average E-bike traffic volume per minute	basic (≤10 E-bikes), low (11-15 E-bikes), medium (16-20 E-bikes), or high (>20 E-bikes)
presence of a traffic controller (traffic policeman or traffic police assistant directing vehicular and pedestrian traffic)	yes or no
type of E-bike	equipped with pedals or otherwise in scooter form
E-bike registration status	registered with registration plate displayed or otherwise unregistered;
E-bikers' gender	male or female
E-bikers' occupation	uniformed couriers or not
carrying passengers	yes or no
carrying oversized cargo (>60 x 40 x 20cm^3)	yes or no
riding in a motor vehicle lane	yes or no
running red lights	yes or no
riding in the opposite direction (i.e., facing oncoming traffic)	yes or no
mobile phone use	yes or no
helmet use	yes or no
wearing leather gloves	yes or no
wearing other motorcycle protective clothing	yes or no

Outcome of interest

1) Speeding was defined as binary, i.e., yes (travel speed >20 km/h) or no (travel speed ≤20km/h), because E-bikes are manufactured to a mandatory Standard (12) with designed maximum travel speed of 20 km/h;

For peer review only - http://bmjopen.bmj.com/site/about/guidelines.xhtml

2) Violation was defined as binary, i.e., yes (at least one of the following road rule violations was observed: carrying passengers, carrying oversized cargo, riding in a motor vehicle lane, running red lights, riding in the opposite direction or using mobile phone), or no;
3) Helmet use was defined as binary, i.e., yes (wearing a motorcycle helmet) or no.

Statistical analysis

Completed observational records were reviewed; and data were entered with double entry. All data analyses were conducted using SAS version 9.2 (SAS Institute, 2002). The inter-observer reliability was assessed using Kappa statistics and agreement reached at least 85% for each pair of observers. Frequencies and proportions of speed reading and observed on-road riding behaviors were calculated where appropriate. We used mixed logistic regression allowing for random selection of observational sites to estimate Odds Ratio (OR) and associated 95% Confidence Interval (CI) for different study outcomes adjusted for observational items. [Table 1] We defined P-values less than 0.05 as statistically significant.

RESULTS

There were a total of 27 observational periods (i.e., 14 in Wuzhong and 13 in Zhangjiagang) for direct observational data collection on 20,647 E-bikes, and 16 periods (i.e., 8 for each district) for speed measurement on 800 E-bikes. The average number of E-bikes per observational period was 729 (range: 103 to 1317) and 803 (range: 552 to 1046) for Wuzhong and Zhangjiagang, respectively.

Table 2 describes the observation results and shows that E-bikes were the dominant transportation means in Wuzhong district. Despite similarities across some

For peer review only - http://bmjopen.bmj.com/site/about/guidelines.xhtml

observational items such as low helmet use (i.e., 2.1% in Wuzhong vs 2.2% in Zhangjiagang) and commonplace carrying of passengers (21.3% vs 20.5%), there were differences in riding violations especially for riding unlicensed E-bikes (3.7% vs 63.7%) [Table 2].

Approximately 83.3% (n=333) and 58.5% (n=234) E-bikers were observed traveling at a speed greater than 20 km/h; approximately 41.3% (n=4,211) and 35.4% (n=3,700) violating at least one of the listed road rules; and 2.5% (n=251) and 3.1% (n=319) using any safety gear in Wuzhong and Zhangjiagang, respectively.

Table 2 Distribution of observational items among E-bike study populations (Wuzhong and Zhangjiagang, China)

		Wuzhong district		Zhangjiagang district	
		with speed measure	without speed measure	with speed measure	without speed measure
Sample size	(n)	400	10202	400	10445
Traffic mix	E-bikes (%)	44.6	46.5	28.2	34.5
	Pedestrians (%)	7.4	9.1	3.0	10.6
	Bicycles (%)	3.5	3.2	9.6	4.7
	Cars (%)	36.2	35.1	57.1	47.6
	Heavy vehicles （%）	8.3	6.2	2.2	2.7
E-bike volume per minute (basic as ≤10 E-bikes, low as 11-15 E-bikes, medium as16-20 E-bikes, or high as >20 E-bikes)	Basic (%)	25.0	40.9	50.0	40.2
	Low (%)	12.5	17.0	37.5	59.8
	Medium (%)	37.5	10.4	12.5	0.0
	High (%)	25.0	31.7	0.0	0.0
Weather	Sunny (%)	75.0	82.8	100.0	69.8
	Cloudy (%)	0.0	9.4	0.0	30.2
	Rainy (%)	25.0	7.8	0.0	0.0
Day of week	Weekday (%)	75.0	75.7	75.0	70.5
Time of day	Morning (%)	37.5	45.7	50.0	39.5
Traffic controller	Yes (%)	n/a	0.0	n/a	25.4
Occupation	Courier (%)	1.8	0.8	1.3	0.5
Gender	Males (%)	69.0	59.8	58.5	51.7
Registration	Yes (%)	94.0	96.3	35.3	36.3
Pedals	Yes (%)	28.0	37.1	53.3	54.3
Carrying passengers	Yes (%)	24.3	21.3	11.0	20.5
Carrying large cargo	Yes (%)	11.5	6.9	15.8	10.1
Riding in a motor vehicle lane	Yes (%)	13.5	3.1	2.3	1.4
Riding opposite direction	Yes (%)	3.0	5.7	25.0	30.8
Mobile phone use	Yes (%)	0.8	0.7	2.0	1.1
Helmet use	Yes (%)	3.3	2.1	5.0	2.2

For peer review only - http://bmjopen.bmj.com/site/about/guidelines.xhtml

For peer review only

Gloves	Yes (%)	0.8	0.4	1.8	0.8
Running red lights	Yes (%)	n/a	16.5	n/a	6.2

No statistically significant evidence indicates the existence of regional variation in terms of elevated odds of speeding, general road rule violations, or lack of helmet use. Compared with female E-bikers, males showed greater ORs of speeding. Reduced ORs of road rule violations or wearing a helmet was associated with riding pedal-equipped E-bikes compared with those in scooter form; whereas elevated ORs of road rule violations were observed among couriers compared with other E-bikers [Table 3].

Table 3. Adjusted Odds Ratios (95% Confidence Interval) for speeding, road rule violations, and helmet use among E-bikers Wuzhong and Zhangjiagang, China

		Speeding	Violations	Safety gear use
Region	Urban	1.14 (0.66-1.99)	1.01 (0.95-1.07)	0.98 (0.84-1.14)
	Rural		reference	
Weather	Sunny	0.29 (0.02-3.58)	0.89 (0.40-1.98)	0.58 (0.19-1. 80)
	Cloudy	--	0.76 (0.31-1.88)	0.55 (0.16-1.98)
	Rainy		reference	
Weekday	Yes	1.73 (0.92-3.25)	0.86 (0.53-1.42)	1.43 (0.72-2.81)
	No		reference	
Time of day	Morning	0.73 (0.47-1.15)	1.07 (0.69-1.65)	1.01 (0.56-1.82)
	Afternoon		reference	
Volume	Basic	0.27 (0.02-3.24)	1.53 (0.77-3.02)	0.86 (0.35-2.14)
	Low	0.50 (0.04-6.11)	1.29 (0.62-2.67)	0.90 (0.34-2.37)
	Medium	0.16 (0.01-2.06)	1.13 (0.36-3.60)	0.66 (0.14-3.02)
	High		reference	
Traffic control	Yes	--	0.76 (0.41-1.38)	1.57 (0.70-3.51)
	No		reference	
Gender	Male	**2.12 (1.50-3.01)**	**1.35 (1.27-1.44)**	0.66 (0.54-0.80)
	Female		reference	
Courier	Yes	0.75 (0.20-2.82)	**5.34 (3.58-7.99)**	**7.21 (4.01-12.98)**
	No		reference	
Registration	Yes	0.96 (0.63-1.44)	**0.82 (0.75-0.88)**	1.18 (0.92-1.52)
	No		reference	
Pedals	Yes	0.79 (0.56-1.12)	**0.66 (0.62-0.70)**	**0.39 (0.32-0.49)**
	No		reference	

Note: Significant results are highlighted in bold

For peer review only - http://bmjopen.bmj.com/site/about/guidelines.xhtml

DISCUSSION

Poor safety practice was commonplace including speeding, road rule violations, and little use of helmets and this did not vary between rural and urban areas. Male E-bikers seemed to bear more risks of speeding and road rule violations. Although couriers were 7 times more likely to wear a helmet when riding an E-bike, they were also 5 times more likely to violate road rules when entering an intersection compared with the other E-bikers. When riding E-bikes with pedals rather than those in scooter form, E-bikers had a lower likelihood of violating road rules and wearing a helmet. These identified safety gaps build on previous evidence[5-10] identifying the need to discourage unsafe practice and encourage safety gear use among E-bikers in China, particularly in the context of China recently joining global action to improve road safety in the next decade.[11]

Consistent with previous studies,[2, 8-10] this study confirmed a range of factors associated with observed E-biker behaviours and revealed the invariant nature of unsafe E-bike riding practice in general. We conducted a similar study during March 2012 in metropolitan Suzhou areas and found 27% of E-bikers violated at least one road rule and 41% used at least one type of safety gear.[10] The current study identified a somewhat higher prevalence of road rule violations (38%) and lower safety gear use (3%). This variation may be explained by the seasonality, e.g., the sharp drop in glove use (from 37% to 0.6%) and helmet use (from 9% to 2%). Zhang et al reported a similar decrease in helmet use among motorcycles in Guangxi during the hot and humid season.[13] The study findings also relate to the previous reports of increasing E-biker fatalities and injuries across mainland China,[4-7] which echoes the call for action to develop policies to improve E-bike safety in China.

For peer review only - http://bmjopen.bmj.com/site/about/guidelines.xhtml

The observed high prevalence of unsafe riding practices implies a need for policy change. Current road rules regulate E-bikes as pedal bicycles that should travel in non-motor vehicle lanes at a maximum speed of 15 km/h,[14] whereas the mandatory Standard for E-bikes specifies a maximum speed of 20 kilometers per hour and a maximum weight of 40 kilograms in addition to requiring a specified braking distance and pedal installment.[12]

However, these specifications may not be widely enforced as most of the electric two-wheelers are not designed and produced in line with the national standards of non-motor vehicles.[15] With regard to those that do comply with the national standards of non-motor vehicles, producers, for marketing purposes, often install the so called "speed limiting devices" on their products. With the speed limiting devices, the maximum speed by which the electric two-wheelers could operate is 20 Km/h as required by the mandatory Standard for E-bikes; whereas, the speed limiting devices are designed and installed in a way that could be easily dismantled by customers themselves or sales persons. Without speed limiting devices, the speed of these electric two-wheelers could effortlessly go beyond 20 Km/h and maybe up to 40 Km/h.[16]

Notably, the *Safety Specifications for Power Driven Vehicles Operating on Roads* defines a motorcycle as being power-driven with the maximum speed exceeding 50 kilometers per hour and a moped with a maximum speed range from 20-50 kilometers per hour.[17] This has the legal implication that any E-bikes (mostly in scooter form) that could travel faster than 20 kilometers per hour should be regulated as motor vehicles by the road rules. Obviously, such conflict between the mandatory Standard

For peer review only - http://bmjopen.bmj.com/site/about/guidelines.xhtml

for E-bikes and road rules might create difficulties for legislative enforcement, including cities where motorcycles are banned.

In addition, the effectiveness of helmets in head injury prevention is well established for both bicyclists [18] and motorcyclists.[19] Regardless of the introduction of compulsory motorcycle helmet use producing a substantial increase in use of helmets among motorcyclists in China,[20] similar regulations were missing for bicyclists and E-bikers. Therefore, road rule revisions to encourage helmet use among E-bikers are urgently needed and should be incorporated into the broad road safety agenda.

As for other cross-sectional observational studies, this study is limited by lack of generalizability to other settings (different regions); possible bias due to unmeasurable confounding (influence of road infrastructure); and possible misclassification due to measurement errors (incorrect speed reading). Thus, care should be taken when interpreting the study findings. To minimize the likelihood of measurement errors, various small-scale pilot studies were conducted to determine the feasibility of the study and to validate the observational instruments. Moreover, this study established a strict quality control scheme and recruited experienced observers who had participated in previous studies using similar techniques.[10] Thus, misclassification may not bias the key findings to an important degree. Nevertheless, the study findings provide new evidence to complement previous findings as to diverse safety issues among E-bikers, and to reinforce the imperative to encourage safety gear use and discourage unsafe on-road practices.

For peer review only - http://bmjopen.bmj.com/site/about/guidelines.xhtml

CONCLUSION

E-bikes are becoming a dominant road transportation means for commuters in China, and they are increasingly used as a sustainable alternative to traditional transportation in other countries because of the low maintenance cost and low polluting mobility. The observed unsafe riding practices signal emerging road safety challenges in China and in similar settings elsewhere. Translating established safety practices such as helmet use and enforcing existing countermeasures such as speed limit devices may be used to improve safety practice among E-bikers. Strong political will is especially needed to leapfrog substantial losses associated with E-bike risk in China without sacrificing mobility needs.

ACKNOWLEDGEMENT

This project has funding support from Bloomberg Philanthropies as part of the Bloomberg Philanthropies Global Road Safety Programme in China. Additional funding was provided by Jiangsu Provincial Centre for Disease Control and Prevention (JKRC2011014). We appreciate special support from the WHO China Office, the Ministry of Health in China, Suzhou Bureau of Health, and Suzhou Bureau of Public Security. We also thank Ziyi Jin, Jianfeng Liu, Qi Zhang, Xianglin Liu, and Yan Lu for their assistance in field work. Wei Du is supported by an NHMRC fellowship. Yilan Liao is supported by the National Natural Science Foundation of China (No. 41101431）.

CONTRIBUTION

Jie Yang and Yihe Hu contributed equally to the study design, research implementation, literature review, data analysis, writing full first draft and contributed

For peer review only - http://bmjopen.bmj.com/site/about/guidelines.xhtml

to subsequent drafts. All the other authors contributed to the conceptual development, data interpretation, critical revision of the first manuscript, and subsequent drafts.

COMPETING INTERESTS: None.

REFERENCES

1 Weinert J, Ma C, Cherry C. The transition to electric bikes in China: history and key reasons for rapid growth. Transportation 2007;34: 301-318

2 Yao L, Wu C. Traffic safety of e-bike riders in China: safety attitudes, risk perception and aberrant riding behaviors. Transportation Research Record: Journal of the Transportation Research Board & Transportation Research Board 91st Annual Meeting 2012: Paper 4599.

3 Hurst D, Wheelock C. Electric two-wheel vehicles (electric bicycles, mopeds, scooters, and motorcycles): market analysis and forecasts. Boulder, Colorado: Pike Research, 2010.

4 Bureau of Traffic Management (2004-2010). China road traffic accidents statistics, 2004 to 2010. Beijing: Ministry of Public Security.

5 Du W, Yang J, Powis B, et al. Epidemiological profile of hospitalised injuries among electric bicycle riders admitted to a rural hospital in Suzhou: a cross sectional study. Injury Prevention 2013 Jun 1. [Epub ahead of print].

6 Duan L, Wu C, Deng X, et al. Road traffic injuries in China, 2006–2008. Chinese Journal of Public Health and Preventive Medicine 2010;21:10-13.

7 Feng Z, Raghuwanshi R, Xu Z, et al. Electric-bicycle-related injury: a rising traffic injury burden in China. Injury Prevention 2010;16: 417-419.

8 Weinert J, Ma C, Yang X, et al. Electric Two-Wheelers in China: Effect on Travel Behavior, Mode Shift, and User Safety Perceptions in a Medium-Sized City. Transportation Research Record: Journal of the Transportation Research Board 2007; 2038:62-68.

9 Wu C, Yao L, Zhang K. The red-light running behavior of electric bike riders and cyclists at urban intersections in China: An observational study. Accident Analysis & Prevention, Available online 2011 July 2.

10 Du W, Yang J, Powis B, et al. Understanding on-road practices of electric bike riders: an observational study in a developed city of China. Accident Analysis and Prevention 2013 Jun 25;59C:319-326. [Epub ahead of print]

11 World Health Organization. Global plan for the decade of action for road safety 2011-2020. Geneva: WHO, 2011. Available from: http://www.who.int/roadsafety/decade_of_action/plan/plan_english.pdf (accessed 10 August 2013)

For peer review only - http://bmjopen.bmj.com/site/about/guidelines.xhtml

12 Standardization Administration of the People’s Republic of China (1999). General technical standards of Electric bicycles (GB17761-1999). Chinese Standards Publisher, 1999.

13 Zhang J, Norton R, Tang K, et al. Motorcycle ownership and injury in China. Int. J. Injury Control and Safety Promotion 2004;11:159-163.

14 China National People’s Congress. Law of the People's Republic of China on road traffic safety. Beijing, 2004.

15 Tsinghua Law School. Aiming at Behavioral Changes: Improving Drink Driving and Speeding Law in China Beijing: Tsinghua University, 2012 (Unpublished Report).

16 http://njrb.njnews.cn/html/2011-03/14/node_69.htm. (accessed 10 August 2013).

17 Standardization Administration of the People’s Republic of China. Safety Specifications for Power Driven Vehicles Operating on Roads (GB7258-2004). Chinese Standards Publisher, 2004.

18 Thompson D, Rivara F, Thompson R. Helmets for preventing head and facial injuries in bicyclists. Cochrane Database of Systematic Reviews (published in Issue 4, 1999).

19 Liu B, Ivers R, Norton R,et al. Helmets for preventing injury in motorcycle riders. Cochrane Database of Systematic Reviews,(published in Issue 1, 2008).

20 Yu J, Ke L, Ivers R, Du W, Senserrick T. Prevalence rates of helmet use among motorcycle riders in a developed region in China. Accident Analysis and Prevention 2011;43:214-219.

For peer review only

For peer review only - http://bmjopen.bmj.com/site/about/guidelines.xhtml

For peer review only

For peer review only - http://bmjopen.bmj.com/site/about/guidelines.xhtml

STROBE 2007 (v4) Statement—Checklist of items that should be included in reports of *cross-sectional studies*

Section/Topic	Item #	Recommendation	Reported on page #
**Title and abstract**	1	(*a*) Indicate the study's design with a commonly used term in the title or the abstract	2
		(*b*) Provide in the abstract an informative and balanced summary of what was done and what was found	2
**Introduction**			
Background/rationale	2	Explain the scientific background and rationale for the investigation being reported	4
Objectives	3	State specific objectives, including any prespecified hypotheses	4
**Methods**			
Study design	4	Present key elements of study design early in the paper	5
Setting	5	Describe the setting, locations, and relevant dates, including periods of recruitment, exposure, follow-up, and data collection	5
Participants	6	(*a*) Give the eligibility criteria, and the sources and methods of selection of participants	5,6
Variables	7	Clearly define all outcomes, exposures, predictors, potential confounders, and effect modifiers. Give diagnostic criteria, if applicable	7
Data sources/ measurement	8*	For each variable of interest, give sources of data and details of methods of assessment (measurement). Describe comparability of assessment methods if there is more than one group	6,7
Bias	9	Describe any efforts to address potential sources of bias	*13*
Study size	10	Explain how the study size was arrived at	7
Quantitative variables	11	Explain how quantitative variables were handled in the analyses. If applicable, describe which groupings were chosen and why	6,7
Statistical methods	12	(*a*) Describe all statistical methods, including those used to control for confounding	8
		(*b*) Describe any methods used to examine subgroups and interactions	8
		(*c*) Explain how missing data were addressed	Not applicable
		(*d*) If applicable, describe analytical methods taking account of sampling strategy	Not applicable
		(*e*) Describe any sensitivity analyses	Not applicable
**Results**			

For peer review only

Participants	13*	(a) Report numbers of individuals at each stage of study—eg numbers potentially eligible, examined for eligibility, confirmed eligible, included in the study, completing follow-up, and analysed	8
		(b) Give reasons for non-participation at each stage	Not applicable
		(c) Consider use of a flow diagram	Not applicable
Descriptive data	14*	(a) Give characteristics of study participants (eg demographic, clinical, social) and information on exposures and potential confounders	8,9
		(b) Indicate number of participants with missing data for each variable of interest	-
Outcome data	15*	Report numbers of outcome events or summary measures	8,9
Main results	16	(*a*) Give unadjusted estimates and, if applicable, confounder-adjusted estimates and their precision (eg, 95% confidence interval). Make clear which confounders were adjusted for and why they were included	10
		(*b*) Report category boundaries when continuous variables were categorized	Yes
		(*c*) If relevant, consider translating estimates of relative risk into absolute risk for a meaningful time period	Not applicable
Other analyses	17	Report other analyses done—eg analyses of subgroups and interactions, and sensitivity analyses	Not applicable
**Discussion**			
Key results	18	Summarise key results with reference to study objectives	11
Limitations	19	Discuss limitations of the study, taking into account sources of potential bias or imprecision. Discuss both direction and magnitude of any potential bias	13
Interpretation	20	Give a cautious overall interpretation of results considering objectives, limitations, multiplicity of analyses, results from similar studies, and other relevant evidence	13
Generalisability	21	Discuss the generalisability (external validity) of the study results	14
**Other information**			
Funding	22	Give the source of funding and the role of the funders for the present study and, if applicable, for the original study on which the present article is based	14

*Give information separately for cases and controls in case-control studies and, if applicable, for exposed and unexposed groups in cohort and cross-sectional studies.

Note: An Explanation and Elaboration article discusses each checklist item and gives methodological background and published examples of transparent reporting. The STROBE checklist is best used in conjunction with this article (freely available on the Web sites of PLoS Medicine at http://www.plosmedicine.org/, Annals of Internal Medicine at http://www.annals.org/, and Epidemiology at http://www.epidem.com/). Information on the STROBE Initiative is available at www.strobe-statement.org.

For peer review only

Unsafe riding practice among electric bikers in Suzhou, China: an observational study

Journal:	*BMJ Open*
Manuscript ID:	bmjopen-2013-003902.R1
Article Type:	Research
Date Submitted by the Author:	04-Nov-2013
Complete List of Authors:	Yang, Jie; Jiangsu provincial centre for disease prevention and control, NCD prevention<br>Hu, Yihe; Suzhou municipal centre for disease prevention and control, NCD prevention<br>Du, Wei; Peking University, Institute of Population Research; University of New South Wales, Neuroscience Research Australia<br>Powis, Brent; International Medical University, School of Postgraduate Studies and Research<br>Ozanne-Smith, Joan; Monash University, Department of Forensic Medicine<br>Liao, Yilan; Chinese Academy of Sciences, Institute of Geographical Sciences and Nature Resources Research<br>Li, Ning; Peking University, Institute of Population Research<br>Wu, Ming; Jiangsu provincial centre for disease prevention and control, NCD prevention
**Primary Subject Heading**:	Epidemiology
Secondary Subject Heading:	Public health, Epidemiology
Keywords:	Electric Bike, Cross Sectional Study, Behavior, Risk Factor Research, Driver

SCHOLARONE™
Manuscripts

Title

Unsafe riding practice among electric bikers in Suzhou, China: an observational study

Corresponding Author

Dr. Ming Wu

Tel/Fax: +86 (25) 8375 9411

Email: wuming@jscdc.cn

Address: Department of Non-communicated Chronic Disease Prevention and Control, Jiangsu Provincial Center for Disease Prevention and Control, Nanjing 210009, China

Authors

Jie Yang, MSc[1*], Yihe Hu, MBBS[2*], Wei Du, PhD[3,4], Brent Powis, PhD[5], Joan Ozanne-Smith, MD[6], Yilan Liao, PhD[7], Ning Li, PhD[3], Ming Wu, PhD[1]

1. Department of Chronic Disease Control, Jiangsu Provincial Centre for Disease Control and Prevention, China
2. Department of Chronic Disease Control, Suzhou Municipal Centre for Disease Control and Prevention, Suzhou, China
3. Institute of Population Research, Peking University, China
4. Neuroscience Research Australia, University of New South Wales, Australia
5. School of Postgraduate Studies and Research, International Medical University, Malaysia
6. Department of Forensic Medicine, Monash University, Australia
7. Institute of Geographical Sciences and Nature Resources Research, Chinese Academy of Sciences, China

* These two authors contributed equally

Key Words: Electric Bike, Cross Sectional Study, Behavior, Risk Factor Research, Driver.

Word count: 2,467 words

For peer review only

Abstract

Background: Increasing Electric bike (E-bike) related fatalities have been increasing rapidly in China and such injuries may be partly attributable to unsafe riding practice

Objectives: To describe potentially unsafe riding behaviors among E-bikers and to investigate factors influencing these practices in China

Methods: In September 2012, a cross-sectional observation study including a speed measurement component was conducted in Wuzhong (an urban District) and Zhangjiagang (a rural District) of Suzhou, Jiangsu Province, China. Hand held radar speed meters were used to read traveling speeds of E-bikes and a pro-forma observation checklist was used to collect data on road riding practice. Mixed-effect logistic regressions were used to calculate adjusted Odds Ratios (ORs) and 95% Confidence Intervals (CIs) for the association between speeding, road rule violations and helmet use and their influencing factors.

Results: Among 800 E-bikes with a speed reading, 70.9% exceeded the designed speed limit of 20 km/h. Among a further 20,647 E-bikers observed, 38.3% did not comply with the road rules when entering intersections; and only 2.2% wore helmets. No regional variation was identified between urban and rural areas. Male was associated with more speeding and road rule violations; whereas riding a pedal-equipped E-bike was associated with less road rule violations and less helmet use.

Conclusion: Unsafe riding practices such as speeding, road rule violations, and lack of helmet use were commonplace among E-bikers, especially among males. The study findings suggest that public awareness improvement, road rule revisions and enforcement are needed to discourage unsafe practices in order to improve E-bike safety in China.

For peer review only

For peer review only - http://bmjopen.bmj.com/site/about/guidelines.xhtml

Article Summary

Article Focus:

- E-biker safety is an emerging public health challenge in China. This study focuses on the unsafe on-road riding behaviours among Chinese E-bikers and factors influencing these practices.

Key Message:

- Direct roadside observation techniques were applied to describe safety practices of E-bikers and hand held radar meters were used to estimate their actual travelling speed.
- The variation of on-road riding behaviours between rural and urban areas was evaluated.
- Factors influencing observed riding behaviours were further investigated.

Strengths and Limitations of the Study:

- In this study, we evaluated how fast E-bikers ride on roads and the possible regional variation of riding behaviours. Furthermore, we investigated factors influencing observed riding behaviours.
- Study findings could provide new evidence to enhance the understanding of on-road riding behaviours among Chinese E-bikers, and to reinforce the imperative to encourage safety gear use and discourage unsafe on-road practices.
- Findings might be limited by lack of generalizability to other settings, possible bias due to unmeasurable confounding, and possible misclassification due to measurement errors.

For peer review only

For peer review only - http://bmjopen.bmj.com/site/about/guidelines.xhtml

INTRODUCTION

In China, the last few years have witnessed the rapid growth of E-bikes (either with pedals or in scooter form) due to increasing mobility demand when public transportation systems are crowded and inconveniently routed.[1] Growing wealth among Chinese also increases affordability of E-bike purchase, normally priced at approximately USD 300. In China, more than 120 million E-bikes were registered by 2011 [2] and globally an estimated 466 million E-bikes are expected to hit the road by 2016. [3]

In China, bicycle use is shifting to E-bike use. Unfortunately, an associated unwanted shift was observed as E-bike related fatalities increased almost 7 times over time from 589 in 2004 to 4,029 in 2010 across the nation, whereas bicycle related fatalities decreased 3 times approximately from 13,655 to 4616 during the same period. [4] Moreover, E-bikers hospitalized for injuries accounted for 57% of serious non-fatal road traffic injuries and 50% of the direct hospitalization cost for all road crash casualties in a rural hospital in Suzhou.[5] Thus, E-biker safety is an emerging public health challenge in China .[5-7]

Whilst unsafe riding practices have been reported among E-bikers using a self-reported survey,[2,8] synchronized video camera recording techniques,[9] and direct roadside observations,[10] no studies have reported on how fast E-bikers ride on roads allowing for possible regional variation, such as rural/urban disparities, commonly observed for other road safety issues. To enhance the understanding of on-road riding behaviors among Chinese E-bikers including evaluation of rural/urban variation, we used direct roadside observation techniques to describe their safety practices and hand held radar meters to estimate their actual traveling speed. We further investigated factors influencing these observed behaviors.

METHODS

We applied a cross-sectional observation research which comprised two components for this study, i.e., observations *with* or *without* speed measurement, which were conducted separately in Suzhou, one of the intervention pilot cities in China for the Bloomberg

For peer review only - http://bmjopen.bmj.com/site/about/guidelines.xhtml

Philanthropies Global Road Safety Programme (This is a multinational programme which take effort to reduce death and serious injury on the roads in ten low-and middle-income countries over five years extending from 2010 to 2014). [11] Suzhou has the sixth highest gross domestic product (GDP) per capita on the Chinese mainland, a resident population of 10 million and at least 2 million E-bikes. [10] The study protocol was approved by the Ethics Committee of Jiangsu Provincial Centre for Disease Control and Prevention.

Field implementation

In Suzhou, two administrative districts, i.e., Wuzhong (urban district) and Zhangjiagang (rural district) were selected to conduct both study components. To select observation sites, a grid was placed over standard maps of Wuzhong and Zhangjiagang, random digits were generated for each grid box for selection and each valid grid box contained at least one intersection having traffic lights. For each randomly selected site, an alternate site was also selected randomly from the grid as a backup. A pilot study was carried out to validate the field feasibility such as having low volumes of E-bikes for speed measurement; at least two-way motor vehicle lanes, pedestrian crossings, and bicycle lanes; enough distance between observation sites so the same E-bikers were unlikely to be observed twice; less likely to interrupt observed behaviors and least likely to increase the crash risk for observers. A total of eight sites (i.e, two from each district for each study component) were randomly selected.

For the speed measurement component, observers concealed themselves at approximately 50 meters from the corner of the intersection and used handheld radar speed meters (Bushnell Velocity 10-1911CM with measurement range of 16-320km/h) [12] to record the speed meter reading; whereas for the study component without speed measurement, the observations were conducted at intersection corners. We randomly selected 4 days in a week including one weekend day (September 11th, 12th, 13th, and 15th) for the speed measurement component and collected information on on-road riding behaviors among E-bikers during another 7-day period (September 17th-23th). The time of day (7.00am–6.59pm) for site observations was set at 2-hour intervals as observational periods and randomly assigned to

For peer review only - http://bmjopen.bmj.com/site/about/guidelines.xhtml

sites.

Four experienced observers were recruited, who had participated in previous roadside E-bike observation studies in other districts in Suzhou.[10] Prior to field implementation, the site observers were trained in specifications of different behaviors, identification of different types of protective items, techniques to observe multiple behaviors especially when an E-bike was moving, and data quality control. Roadside pilot observations and regular on-site audits were conducted to ensure the safety guidelines and accurate and appropriate implementation of the data collection process.

The observers worked in pairs, to observe oncoming E-bikes in ascending distance order. Data items were collected on a pro-forma checklist including type of E-bike (with bike pedals or in scooter form), registration status, rider's gender, couriers or not (In China, many couriers are required to wear uniforms when working and companies provide them uniforms with own logos; although uniforms are in different styles, it's easy to differentiate couriers from normal E-bikers), carrying passengers, carrying oversized cargo (estimated >60 x 40 x 20cm^3, the size of normal airlines carry-on luggage), riding in a motor vehicle lane, running red lights, riding in the opposite direction (i.e., facing oncoming traffic), using mobile phone, using helmet, wearing leather gloves, and wearing other motorcycle protective clothing. Weather, day of week, time of day, average E-bike traffic volume per minute, presence of a traffic controller (traffic policeman or traffic police assistant directing vehicular and pedestrian traffic) were recorded on separate data collection forms.[Table 1] Regarding the speed measurement component, for every tenth E-bike, the second observer recorded the radar speed reading during the speed measurement observations.

Outcome of interest

1) Speeding was defined as binary, i.e., yes (travel speed >20 km/h) or no (travel speed ≤20km/h), because E-bikes are manufactured to a mandatory Standard [13] with designed maximum travel speed of 20 km/h;
2) Violation was defined as binary, i.e., yes (at least one of the following road rule violations

For peer review only - http://bmjopen.bmj.com/site/about/guidelines.xhtml

was observed: carrying passengers, carrying oversized cargo, riding in a motor vehicle lane, running red lights, riding in the opposite direction or using mobile phone), or no;

3) Helmet use was defined as binary, i.e., yes (wearing a motorcycle helmet) or no.

Table 1. Observational item categorization

Observational items	Categorization
weather	sunny, cloudy, or rainy
day of week	weekday or weekend
time of day	morning or afternoon
average E-bike traffic volume per minute	basic (≤10 E-bikes), low (11-15 E-bikes), medium (16-20 E-bikes), or high (>20 E-bikes)
presence of a traffic controller (traffic policeman or traffic police assistant directing vehicular and pedestrian traffic)	yes or no
type of E-bike	equipped with pedals or otherwise in scooter form
E-bike registration status	registered with registration plate displayed or otherwise unregistered;
E-bikers' gender	male or female
E-bikers' occupation	couriers or not
carrying passengers	yes or no
carrying oversized cargo (>60 x 40 x 20cm^3)	yes or no
riding in a motor vehicle lane	yes or no
running red lights	yes or no
riding in the opposite direction (i.e., facing oncoming traffic)	yes or no
mobile phone use	yes or no
helmet use	yes or no
wearing leather gloves	yes or no
wearing other motorcycle protective clothing	yes or no

Statistical analysis

Completed observational records were reviewed; and data were entered with double entry. All data analyses were conducted using SAS version 9.2 (SAS Institute, 2002). The inter-observer reliability was assessed using Kappa statistics and agreement reached at least 85% for each pair of observers. Frequencies and proportions of speed reading and observed on-road riding behaviors were calculated where appropriate. We used mixed logistic regression allowing for random selection of observational sites to estimate Odds Ratio (OR) and associated 95% Confidence Interval (CI) for different study outcomes adjusted for observational items. [Table 1] We defined P-values less than 0.05 as statistically significant.

For peer review only - http://bmjopen.bmj.com/site/about/guidelines.xhtml

RESULTS

There were a total of 27 observational periods (i.e., 14 in Wuzhong and 13 in Zhangjiagang) for direct observational data collection on 20,647 E-bikes, and 16 periods (i.e., 8 for each district) for speed measurement on 800 E-bikes. The average number of E-bikes per observational period was 729 (range: 103 to 1317) and 803 (range: 552 to 1046) for Wuzhong and Zhangjiagang, respectively.

Table 2 Distribution of observational items among E-bike study populations

		Wuzhong (Urban district)		Zhangjiagang Rural district)	
		with speed measure	without speed measure	with speed measure	without speed measure
Sample size	(n)	400	10202	400	10445
Traffic mix	E-bikes (%)	44.6	46.5	28.2	34.5
	Pedestrians (%)	7.4	9.1	3.0	10.6
	Bicycles (%)	3.5	3.2	9.6	4.7
	Cars (%)	36.2	35.1	57.1	47.6
	Heavy vehicles （%）	8.3	6.2	2.2	2.7
E-bike volume per minute (basic as ≤10 E-bikes, low as 11-15 E-bikes, medium as16-20 E-bikes, or high as >20 E-bikes)	Basic (%)	25.0	40.9	50.0	40.2
	Low (%)	12.5	17.0	37.5	59.8
	Medium (%)	37.5	10.4	12.5	0.0
	High (%)	25.0	31.7	0.0	0.0
Weather	Sunny (%)	75.0	82.8	100.0	69.8
	Cloudy (%)	0.0	9.4	0.0	30.2
	Rainy (%)	25.0	7.8	0.0	0.0
Day of week	Weekday (%)	75.0	75.7	75.0	70.5
Time of day	Morning (%)	37.5	45.7	50.0	39.5
Traffic controller	Yes (%)	Not applicable	0.0	Not applicable	25.4
Occupation	Courier (%)	1.8	0.8	1.3	0.5
Gender	Males (%)	69.0	59.8	58.5	51.7
Registration	Yes (%)	94.0	96.3	35.3	36.3
Pedals	Yes (%)	28.0	37.1	53.3	54.3
Carrying passengers	Yes (%)	24.3	21.3	11.0	20.5
Carrying large cargo	Yes (%)	11.5	6.9	15.8	10.1
Riding in a motor vehicle lane	Yes (%)	13.5	3.1	2.3	1.4
Riding opposite direction	Yes (%)	3.0	5.7	25.0	30.8
Mobile phone use	Yes (%)	0.8	0.7	2.0	1.1
Helmet use	Yes (%)	3.3	2.1	5.0	2.2
Gloves	Yes (%)	0.8	0.4	1.8	0.8
Running red lights	Yes (%)	Not applicable	16.5	Not applicable	6.2

For peer review only - http://bmjopen.bmj.com/site/about/guidelines.xhtml

For peer review only

Table 2 describes the observation results and shows that E-bikes were the dominant transportation means in Wuzhong district. Despite similarities across some observational items such as low helmet use (i.e., 2.1% in Wuzhong vs 2.2% in Zhangjiagang) and commonplace carrying of passengers (21.3% vs 20.5%), there were differences in riding violations especially for riding licensed E-bikes (96.3% vs 36.3%) [Table 2].

Approximately 83.3% (n=333) and 58.5% (n=234) E-bikers were observed traveling at a speed greater than 20 km/h; approximately 41.3% (n=4,211) and 35.4% (n=3,700) violating at least one of the listed road rules; and 2.5% (n=251) and 3.1% (n=319) using any safety gear in Wuzhong and Zhangjiagang, respectively.

No statistically significant evidence indicates the existence of regional variation in terms of elevated odds of speeding, general road rule violations, or lack of helmet use. Compared with female E-bikers, males showed greater ORs of speeding (OR=2.12, 95%CI=1.50-3.01) and violation (OR=1.35, 95%CI=1.27-1.44). Reduced ORs of road rule violations (OR=0.66, 95%CI=0.62-0.70) and wearing a helmet (OR=0.39, 95%CI=0.32-0.49) were found to be associated with riding pedal-equipped E-bikes compared with those in scooter form; whereas the highest elevated ORs of Helmet use (OR=7.21,95%CI=4.01-12.98) and road rule violations (OR=5.34, 95%CI=3.58-7.99) were observed among couriers compared with other E-bikers [Table 3].

For peer review only

Table 3. Adjusted Odds Ratios (95% Confidence Intervals) for speeding, road rule violations, and helmet use among E-bikers *

		Speeding N=800	Violations N=20,647	Helmet use N=20,647
Region	Urban	1.14 (0.66-1.99)	1.01 (0.95-1.07)	0.98 (0.84-1.14)
	Rural		reference	
Weather	Sunny	0.29 (0.02-3.58)	0.89 (0.40-1.98)	0.58 (0.19-1. 80)
	Cloudy	--	0.76 (0.31-1.88)	0.55 (0.16-1.98)
	Rainy		reference	
Weekday	Yes	1.73 (0.92-3.25)	0.86 (0.53-1.42)	1.43 (0.72-2.81)
	No		reference	
Time of day	Morning	0.73 (0.47-1.15)	1.07 (0.69-1.65)	1.01 (0.56-1.82)
	Afternoon		reference	
Volume	Basic	0.27 (0.02-3.24)	1.53 (0.77-3.02)	0.86 (0.35-2.14)
	Low	0.50 (0.04-6.11)	1.29 (0.62-2.67)	0.90 (0.34-2.37)
	Medium	0.16 (0.01-2.06)	1.13 (0.36-3.60)	0.66 (0.14-3.02)
	High		reference	
Traffic control	Yes	--	0.76 (0.41-1.38)	1.57 (0.70-3.51)
	No		reference	
Gender	Male	**2.12 (1.50-3.01)**	**1.35 (1.27-1.44)**	0.66 (0.54-0.80)
	Female		reference	
Courier	Yes	0.75 (0.20-2.82)	**5.34 (3.58-7.99)**	**7.21 (4.01-12.98)**
	No		reference	
Registration	Yes	0.96 (0.63-1.44)	**0.82 (0.75-0.88)**	1.18 (0.92-1.52)
	No		reference	
Pedals	Yes	0.79 (0.56-1.12)	**0.66 (0.62-0.70)**	**0.39 (0.32-0.49)**
	No		reference	

* The adjusting variables were observational items showed in Table1.
Note: Significant results are highlighted in bold

DISCUSSION

Poor safety practice was commonplace including speeding, road rule violations, and little use of helmets and this did not vary between rural and urban areas. Male E-bikers seemed to bear more risks of speeding and road rule violations. Although couriers were 7 times more likely to wear a helmet when riding an E-bike, they were also 5 times more likely to violate road rules when entering an intersection compared with the other E-bikers. When riding E-bikes with pedals rather than those in scooter form, E-bikers had a lower likelihood of violating road rules and wearing a helmet. These identified safety gaps build on previous evidence[5-10] identifying the need to discourage unsafe practice and encourage safety gear use among

For peer review only - http://bmjopen.bmj.com/site/about/guidelines.xhtml

E-bikers in China, particularly in the context of China recently joining global action to improve road safety in the next decade.[11]

Consistent with previous studies,[2, 8-10] this study confirmed a range of factors associated with observed E-biker behaviours and revealed the invariant nature of unsafe E-bike riding practice in general. We conducted a similar study during March 2012 in metropolitan Suzhou areas and found 27% of E-bikers violated at least one road rule and 41% used at least one type of safety gear.[10] The current study identified a somewhat higher prevalence of road rule violations (38%) and lower safety gear use (3%). This variation may be explained by the seasonality, e.g., the sharp drop in glove use (from 37% to 0.6%) and helmet use (from 9% to 2%). Zhang et al reported a similar decrease in helmet use among motorcycles in Guangxi during the hot and humid season.[14] The study findings also relate to the previous reports of increasing E-biker fatalities and injuries across mainland China,[4-7] which echoes the call for action to develop policies to improve E-bike safety in China.

The observed high prevalence of unsafe riding practices implies a need for policy change. Current road rules regulate E-bikes as pedal bicycles that should travel in non-motor vehicle lanes at a maximum speed of 15 km/h,[15] whereas the mandatory Standard for E-bikes specifies a maximum speed of 20 kilometers per hour and a maximum weight of 40 kilograms in addition to requiring a specified braking distance and pedal installment.[13]

However, these specifications may not be widely enforced as most of the electric two-wheelers are not designed and produced in line with the national standards of non-motor vehicles.[16] With regard to those that do comply with the national standards of non-motor vehicles, producers, for marketing purposes, often install the so called "speed limiting devices" on their products. With the speed limiting devices, the maximum speed by which the electric two-wheelers could operate is 20 Km/h as required by the mandatory Standard for E-bikes; whereas, the speed limiting devices are designed and installed in a way that could be easily dismantled by customers themselves or sales persons. Without speed limiting devices,

For peer review only - http://bmjopen.bmj.com/site/about/guidelines.xhtml

the speed of these electric two-wheelers could effortlessly go beyond 20 Km/h and maybe up to 40 Km/h [17].

Notably, the *Safety Specifications for Power Driven Vehicles Operating on Roads* defines a motorcycle as being power-driven with the maximum speed exceeding 50 kilometers per hour and a moped with a maximum speed range from 20-50 kilometers per hour.[18] This has the legal implication that any E-bikes (mostly in scooter form) that could travel faster than 20 kilometers per hour should be regulated as motor vehicles by the road rules. Obviously, such conflict between the mandatory Standard for E-bikes and road rules might create difficulties for legislative enforcement, including cities where motorcycles are banned.

In addition, the effectiveness of helmets in head injury prevention is well established for both bicyclists [19] and motorcyclists.[20] Regardless of the introduction of compulsory motorcycle helmet use producing a substantial increase in use of helmets among motorcyclists in China,[21] similar regulations were missing for bicyclists and E-bikers. Therefore, road rule revisions to encourage helmet use among E-bikers are urgently needed and should be incorporated into the broad road safety agenda.

To our best knowledge, no international E-bikers` riding practice studies were conducted before. Compared with other cross-sectional observational studies, this study is limited by lack of generalizability to other settings (different regions); possible bias due to unmeasurable confounding (influence of road infrastructure); and possible misclassification due to measurement errors (incorrect speed reading). Thus, care should be taken when interpreting the study findings. To minimize the likelihood of measurement errors, various small-scale pilot studies were conducted to determine the feasibility of the study and to validate the observational instruments. Moreover, this study established a strict quality control scheme and recruited experienced observers who had participated in previous studies using similar techniques.[10] Thus, misclassification may not bias the key findings to an important degree.

For peer review only - http://bmjopen.bmj.com/site/about/guidelines.xhtml

For peer review only

Nevertheless, the study findings provide new evidence to complement previous findings as to diverse safety issues among E-bikers, and to reinforce the imperative to encourage safety gear use and discourage unsafe on-road practices.

CONCLUSION

E-bikes are becoming a dominant road transportation means for commuters in China, and they are increasingly used as a sustainable alternative to traditional transportation in other countries because of the low maintenance cost and low polluting mobility. The observed unsafe riding practices signal emerging road safety challenges in China and in similar settings elsewhere. Translating established safety practices such as helmet use and enforcing existing countermeasures such as speed limit devices may be used to improve safety practice among E-bikers. Strong political will is especially needed to leapfrog substantial losses associated with E-bike risk in China without sacrificing mobility needs.

ACKNOWLEDGEMENT

This project has funding support from Bloomberg Philanthropies as part of the Bloomberg Philanthropies Global Road Safety Programme in China. Additional funding was provided by Jiangsu Provincial Centre for Disease Control and Prevention (JKRC2011014). We appreciate special support from the WHO China Office, the Ministry of Health in China, Suzhou Bureau of Health, and Suzhou Bureau of Public Security. We also thank Ziyi Jin, Jianfeng Liu, Qi Zhang, Xianglin Liu, and Yan Lu for their assistance in field work. Wei Du is supported by an NHMRC fellowship. Yilan Liao is supported by the National Natural Science Foundation of China (No. 41101431）.

CONTRIBUTION

Jie Yang and Yihe Hu contributed equally to the study design, research implementation, literature review, data analysis, writing full first draft and contributed to subsequent drafts. All the other authors contributed to the conceptual development, data interpretation, critical

For peer review only - http://bmjopen.bmj.com/site/about/guidelines.xhtml

revision of the first manuscript, and subsequent drafts.

COMPETING INTERESTS: There is no conflict of interest.

REFERENCES

1 Weinert J, Ma C, Cherry C. The transition to electric bikes in China: history and key reasons for rapid growth. Transportation 2007;34: 301-318

2 2Yao L, Wu C. Traffic safety of e-bike riders in China: safety attitudes, risk perception and aberrant riding behaviors. Transportation Research Record: Journal of the Transportation Research Board & Transportation Research Board 91st Annual Meeting 2012: Paper 4599.

3 3Hurst D, Wheelock C. Electric two-wheel vehicles (electric bicycles, mopeds, scooters, and motorcycles): market analysis and forecasts. Boulder, Colorado: Pike Research, 2010.

4 Bureau of Traffic Management (2004-2010). China road traffic accidents statistics, 2004 to 2010. Beijing: Ministry of Public Security.

5 Du W, Yang J, Powis B, et al. Epidemiological profile of hospitalised injuries among electric bicycle riders admitted to a rural hospital in Suzhou: a cross sectional study. Injury Prevention 2013 Jun 1. [Epub ahead of print].

6 Duan L, Wu C, Deng X, et al. Road traffic injuries in China, 2006–2008. Chinese Journal of Public Health and Preventive Medicine 2010;21:10-13.

7 Feng Z, Raghuwanshi R, Xu Z, et al. Electric-bicycle-related injury: a rising traffic injury burden in China. Injury Prevention 2010;16: 417-419.

8 Weinert J, Ma C, Yang X, et al. Electric Two-Wheelers in China: Effect on Travel Behavior, Mode Shift, and User Safety Perceptions in a Medium-Sized City. Transportation Research Record: Journal of the Transportation Research Board 2007; 2038:62-68.

9 Wu C, Yao L, Zhang K. The red-light running behavior of electric bike riders and cyclists at urban intersections in China: An observational study. Accident Analysis & Prevention, Available online 2011 July 2.

10 Du W, Yang J, Powis B, et al. Understanding on-road practices of electric bike riders: an observational study in a developed city of China. Accident Analysis and Prevention 2013 Jun 25;59C:319-326. [Epub ahead of print]

11 World Health Organization. Global plan for the decade of action for road safety 2011-2020. Geneva: WHO, 2011. Available from:http://www.who.int/roadsafety/decade_of_action/plan/plan_english.pdf (accessed 10 Aug 2013)

12 http://www.bushnell.com/all-products/outdoor-technology/velocity-speed-gun. (accessed 1 Nov 2013)

13 Standardization Administration of the People's Republic of China (1999). General technical

For peer review only - http://bmjopen.bmj.com/site/about/guidelines.xhtml

standards of Electric bicycles (GB17761-1999). Chinese Standards Publisher, 1999.

14 Zhang J, Norton R, Tang K, et al. Motorcycle ownership and injury in China. Int. J. Injury Control and Safety Promotion 2004;11:159-163.

15 China National People's Congress. Law of the People's Republic of China on road traffic safety. Beijing, 2004.

16 Tsinghua Law School. Aiming at Behavioral Changes: Improving Drink Driving and Speeding Law in China Beijing: Tsinghua University, 2012 (Unpublished Report).

17 http://njrb.njnews.cn/html/2011-03/14/node_69.htm. (accessed 10 August 2013).

18 Standardization Administration of the People's Republic of China. Safety Specifications for Power Driven Vehicles Operating on Roads (GB7258-2004). Chinese Standards Publisher, 2004.

19 Thompson D, Rivara F, Thompson R. Helmets for preventing head and facial injuries in bicyclists. Cochrane Database of Systematic Reviews (published in Issue 4, 1999).

20 Liu B, Ivers R, Norton R,et al. Helmets for preventing injury in motorcycle riders. Cochrane Database of Systematic Reviews,(published in Issue 1, 2008).

21 Yu J, Ke L, Ivers R, Du W, Senserrick T. Prevalence rates of helmet use among motorcycle riders in a developed region in China. Accident Analysis and Prevention 2011;43:214-219.

For peer review only

For peer review only - http://bmjopen.bmj.com/site/about/guidelines.xhtml

Title

Unsafe riding practice among electric bikers in Suzhou, China: an observational study

Corresponding Author

Dr. Ming Wu
Tel/Fax: +86 (25) 8375 9411
Email: wuming@jscdc.cn
Address: Department of Non-communicated Chronic Disease Prevention and Control, Jiangsu Provincial Center for Disease Prevention and Control, Nanjing 210009, China

Authors

Jie Yang, MSc[1*], Yihe Hu, MBBS[2*], Wei Du, PhD[3,4], Brent Powis, PhD[5], Joan Ozanne-Smith, MD[6], Yilan Liao, PhD[7], Ning Li, PhD[3], Ming Wu, PhD[1]

1. Department of Chronic Disease Control, Jiangsu Provincial Centre for Disease Control and Prevention, China
2. Department of Chronic Disease Control, Suzhou Municipal Centre for Disease Control and Prevention, Suzhou, China
3. Institute of Population Research, Peking University, China
4. Neuroscience Research Australia, University of New South Wales, Australia
5. School of Postgraduate Studies and Research, International Medical University, Malaysia
6. Department of Forensic Medicine, Monash University, Australia
7. Institute of Geographical Sciences and Nature Resources Research, Chinese Academy of Sciences, China

* These two authors contributed equally

Key Words: Electric Bike, Cross Sectional Study, Behavior, Risk Factor Research, Driver.

Word count: 2,467 words

For peer review only - http://bmjopen.bmj.com/site/about/guidelines.xhtml

Abstract

Background: Increasing Electric bike (E-bike) related fatalities have been increasing rapidly in China and such injuries may be partly attributable to unsafe riding practice

Objectives: To describe potentially unsafe riding behaviors among E-bikers and to investigate factors influencing these practices in China

Methods: In September 2012, a cross-sectional observation study including a speed measurement component was conducted in Wuzhong (an urban District) and Zhangjiagang (a rural District) of Suzhou, Jiangsu Province, China. Hand held radar speed meters were used to read traveling speeds of E-bikes and a pro-forma observation checklist was used to collect data on road riding practice. Mixed-effect logistic regressions were used to calculate adjusted Odds Ratios (ORs) and 95% Confidence Intervals (CIs) for the association between speeding, road rule violations and helmet use and their influencing factors.

Results: Among 800 E-bikes with a speed reading, 70.9% exceeded the designed speed limit of 20 km/h. Among a further 20,647 E-bikers observed, 38.3% did not comply with the road rules when entering intersections; and only 2.2% wore helmets. No regional variation was identified between urban and rural areas. Male was associated with more speeding and road rule violations; whereas riding a pedal-equipped E-bike was associated with less road rule violations and less helmet use.

Conclusion: Unsafe riding practices such as speeding, road rule violations, and lack of helmet use were commonplace among E-bikers, especially among males. The study findings suggest that public awareness improvement, road rule revisions and enforcement are needed to discourage unsafe practices in order to improve E-bike safety in China.

For peer review only - http://bmjopen.bmj.com/site/about/guidelines.xhtml

Article Summary

Article Focus:

- E-biker safety is an emerging public health challenge in China. This study focuses on the unsafe on-road riding behaviours among Chinese E-bikers and factors influencing these practices.

Key Message:

- Direct roadside observation techniques were applied to describe safety practices of E-bikers and hand held radar meters were used to estimate their actual travelling speed.
- The variation of on-road riding behaviours between rural and urban areas was evaluated.
- Factors influencing observed riding behaviours were further investigated.

Strengths and Limitations of the Study:

- In this study, we evaluated how fast E-bikers ride on roads and the possible regional variation of riding behaviours. Furthermore, we investigated factors influencing observed riding behaviours.
- Study findings could provide new evidence to enhance the understanding of on-road riding behaviours among Chinese E-bikers, and to reinforce the imperative to encourage safety gear use and discourage unsafe on-road practices.
- Findings might be limited by lack of generalizability to other settings, possible bias due to unmeasurable confounding, and possible misclassification due to measurement errors.

For peer review only - http://bmjopen.bmj.com/site/about/guidelines.xhtml

INTRODUCTION

In China, the last few years have witnessed the rapid growth of E-bikes (either with pedals or in scooter form) due to increasing mobility demand when public transportation systems are crowded and inconveniently routed.[1] Growing wealth among Chinese also increases affordability of E-bike purchase, normally priced at approximately USD 300. In China, more than 120 million E-bikes were registered by 2011 [2] and globally an estimated 466 million E-bikes are expected to hit the road by 2016. [3]

In China, bicycle use is shifting to E-bike use. Unfortunately, an associated unwanted shift was observed as E-bike related fatalities increased almost 7 times over time from 589 in 2004 to 4,029 in 2010 across the nation, whereas bicycle related fatalities decreased 3 times approximately from 13,655 to 4616 during the same period. [4] Moreover, E-bikers hospitalized for injuries accounted for 57% of serious non-fatal road traffic injuries and 50% of the direct hospitalization cost for all road crash casualties in a rural hospital in Suzhou.[5] Thus, E-biker safety is an emerging public health challenge in China .[5-7]

Whilst unsafe riding practices have been reported among E-bikers using a self-reported survey,[2,8] synchronized video camera recording techniques,[9] and direct roadside observations,[10] no studies have reported on how fast E-bikers ride on roads allowing for possible regional variation, such as rural/urban disparities, commonly observed for other road safety issues. To enhance the understanding of on-road riding behaviors among Chinese E-bikers including evaluation of rural/urban variation, we used direct roadside observation techniques to describe their safety practices and hand held radar meters to estimate their actual traveling speed. We further investigated factors influencing these observed behaviors.

METHODS

We applied a cross-sectional observation research which comprised two components for this study, i.e., observations *with* or *without* speed measurement, which were conducted separately in Suzhou, one of the intervention pilot cities in China for the Bloomberg

For peer review only - http://bmjopen.bmj.com/site/about/guidelines.xhtml

Philanthropies Global Road Safety Programme (This is a multinational programme which take effort to reduce death and serious injury on the roads in ten low-and middle-income countries over five years extending from 2010 to 2014). [11] Suzhou has the sixth highest gross domestic product (GDP) per capita on the Chinese mainland, a resident population of 10 million and at least 2 million E-bikes. [10] The study protocol was approved by the Ethics Committee of Jiangsu Provincial Centre for Disease Control and Prevention.

Field implementation

In Suzhou, two administrative districts, i.e., Wuzhong (urban district) and Zhangjiagang (rural district) were selected to conduct both study components. To select observation sites, a grid was placed over standard maps of Wuzhong and Zhangjiagang, random digits were generated for each grid box for selection and each valid grid box contained at least one intersection having traffic lights. For each randomly selected site, an alternate site was also selected randomly from the grid as a backup. A pilot study was carried out to validate the field feasibility such as having low volumes of E-bikes for speed measurement; at least two-way motor vehicle lanes, pedestrian crossings, and bicycle lanes; enough distance between observation sites so the same E-bikers were unlikely to be observed twice; less likely to interrupt observed behaviors and least likely to increase the crash risk for observers. A total of eight sites (i.e, two from each district for each study component) were randomly selected.

For the speed measurement component, observers concealed themselves at approximately 50 meters from the corner of the intersection and used handheld radar speed meters (Bushnell Velocity 10-1911CM with measurement range of 16-320km/h) [12] to record the speed meter reading; whereas for the study component without speed measurement, the observations were conducted at intersection corners. We randomly selected 4 days in a week including one weekend day (September 11th, 12th, 13th, and 15th) for the speed measurement component and collected information on on-road riding behaviors among E-bikers during another 7-day period (September 17th-23th). The time of day (7.00am–6.59pm) for site observations was set at 2-hour intervals as observational periods and randomly assigned to

For peer review only - http://bmjopen.bmj.com/site/about/guidelines.xhtml

sites.

Four experienced observers were recruited, who had participated in previous roadside E-bike observation studies in other districts in Suzhou.[10] Prior to field implementation, the site observers were trained in specifications of different behaviors, identification of different types of protective items, techniques to observe multiple behaviors especially when an E-bike was moving, and data quality control. Roadside pilot observations and regular on-site audits were conducted to ensure the safety guidelines and accurate and appropriate implementation of the data collection process.

The observers worked in pairs, to observe oncoming E-bikes in ascending distance order. Data items were collected on a pro-forma checklist including type of E-bike (with bike pedals or in scooter form), registration status, rider's gender, couriers or not (In China, many couriers are required to wear uniforms when working and companies provide them uniforms with own logos; although uniforms are in different styles, it's easy to differentiate couriers from normal E-bikers), carrying passengers, carrying oversized cargo (estimated >60 x 40 x 20cm^3, the size of normal airlines carry-on luggage), riding in a motor vehicle lane, running red lights, riding in the opposite direction (i.e., facing oncoming traffic), using mobile phone, using helmet, wearing leather gloves, and wearing other motorcycle protective clothing. Weather, day of week, time of day, average E-bike traffic volume per minute, presence of a traffic controller (traffic policeman or traffic police assistant directing vehicular and pedestrian traffic) were recorded on separate data collection forms.[Table 1] Regarding the speed measurement component, for every tenth E-bike, the second observer recorded the radar speed reading during the speed measurement observations.

Outcome of interest

1) Speeding was defined as binary, i.e., yes (travel speed >20 km/h) or no (travel speed ≤20km/h), because E-bikes are manufactured to a mandatory Standard [13] with designed maximum travel speed of 20 km/h;
2) Violation was defined as binary, i.e., yes (at least one of the following road rule violations

For peer review only - http://bmjopen.bmj.com/site/about/guidelines.xhtml

was observed: carrying passengers, carrying oversized cargo, riding in a motor vehicle lane, running red lights, riding in the opposite direction or using mobile phone), or no;

3) Helmet use was defined as binary, i.e., yes (wearing a motorcycle helmet) or no.

Table 1. Observational item categorization

Observational items	Categorization
weather	sunny, cloudy, or rainy
day of week	weekday or weekend
time of day	morning or afternoon
average E-bike traffic volume per minute	basic (≤10 E-bikes), low (11-15 E-bikes), medium (16-20 E-bikes), or high (>20 E-bikes)
presence of a traffic controller (traffic policeman or traffic police assistant directing vehicular and pedestrian traffic)	yes or no
type of E-bike	equipped with pedals or otherwise in scooter form
E-bike registration status	registered with registration plate displayed or otherwise unregistered;
E-bikers' gender	male or female
E-bikers' occupation	couriers or not
carrying passengers	yes or no
carrying oversized cargo (>60 x 40 x 20cm^3)	yes or no
riding in a motor vehicle lane	yes or no
running red lights	yes or no
riding in the opposite direction (i.e., facing oncoming traffic)	yes or no
mobile phone use	yes or no
helmet use	yes or no
wearing leather gloves	yes or no
wearing other motorcycle protective clothing	yes or no

For peer review only

Statistical analysis

Completed observational records were reviewed; and data were entered with double entry. All data analyses were conducted using SAS version 9.2 (SAS Institute, 2002). The inter-observer reliability was assessed using Kappa statistics and agreement reached at least 85% for each pair of observers. Frequencies and proportions of speed reading and observed on-road riding behaviors were calculated where appropriate. We used mixed logistic regression allowing for random selection of observational sites to estimate Odds Ratio (OR) and associated 95% Confidence Interval (CI) for different study outcomes adjusted for observational items. [Table 1] We defined P-values less than 0.05 as statistically significant.

RESULTS

There were a total of 27 observational periods (i.e., 14 in Wuzhong and 13 in Zhangjiagang) for direct observational data collection on 20,647 E-bikes, and 16 periods (i.e., 8 for each district) for speed measurement on 800 E-bikes. The average number of E-bikes per observational period was 729 (range: 103 to 1317) and 803 (range: 552 to 1046) for Wuzhong and Zhangjiagang, respectively.

For peer review only

Table 2 Distribution of observational items among E-bike study populations

		Wuzhong (Urban district)		Zhangjiagang Rural district)	
		with speed measure	without speed measure	with speed measure	without speed measure
Sample size	(n)	400	10202	400	10445
Traffic mix	E-bikes (%)	44.6	46.5	28.2	34.5
	Pedestrians (%)	7.4	9.1	3.0	10.6
	Bicycles (%)	3.5	3.2	9.6	4.7
	Cars (%)	36.2	35.1	57.1	47.6
	Heavy vehicles （%）	8.3	6.2	2.2	2.7
E-bike volume per minute (basic as ≤10 E-bikes, low as 11-15 E-bikes, medium as16-20 E-bikes, or high as >20 E-bikes)	Basic (%)	25.0	40.9	50.0	40.2
	Low (%)	12.5	17.0	37.5	59.8
	Medium (%)	37.5	10.4	12.5	0.0
	High (%)	25.0	31.7	0.0	0.0
Weather	Sunny (%)	75.0	82.8	100.0	69.8
	Cloudy (%)	0.0	9.4	0.0	30.2
	Rainy (%)	25.0	7.8	0.0	0.0
Day of week	Weekday (%)	75.0	75.7	75.0	70.5
Time of day	Morning (%)	37.5	45.7	50.0	39.5
Traffic controller	Yes (%)	Not applicable	0.0	Not applicable	25.4
Occupation	Courier (%)	1.8	0.8	1.3	0.5
Gender	Males (%)	69.0	59.8	58.5	51.7
Registration	Yes (%)	94.0	96.3	35.3	36.3
Pedals	Yes (%)	28.0	37.1	53.3	54.3
Carrying passengers	Yes (%)	24.3	21.3	11.0	20.5
Carrying large cargo	Yes (%)	11.5	6.9	15.8	10.1
Riding in a motor vehicle lane	Yes (%)	13.5	3.1	2.3	1.4
Riding opposite direction	Yes (%)	3.0	5.7	25.0	30.8
Mobile phone use	Yes (%)	0.8	0.7	2.0	1.1
Helmet use	Yes (%)	3.3	2.1	5.0	2.2
Gloves	Yes (%)	0.8	0.4	1.8	0.8
Running red lights	Yes (%)	Not applicable	16.5	Not applicable	6.2

For peer review only - http://bmjopen.bmj.com/site/about/guidelines.xhtml

Table 2 describes the observation results and shows that E-bikes were the dominant transportation means in Wuzhong district. Despite similarities across some observational items such as low helmet use (i.e., 2.1% in Wuzhong vs 2.2% in Zhangjiagang) and commonplace carrying of passengers (21.3% vs 20.5%), there were differences in riding violations especially for riding licensed E-bikes (96.3% vs 36.3%) [Table 2].

Approximately 83.3% (n=333) and 58.5% (n=234) E-bikers were observed traveling at a speed greater than 20 km/h; approximately 41.3% (n=4,211) and 35.4% (n=3,700) violating at least one of the listed road rules; and 2.5% (n=251) and 3.1% (n=319) using any safety gear in Wuzhong and Zhangjiagang, respectively.

No statistically significant evidence indicates the existence of regional variation in terms of elevated odds of speeding, general road rule violations, or lack of helmet use. Compared with female E-bikers, males showed greater ORs of speeding (OR=2.12, 95%CI=1.50-3.01) and violation (OR=1.35, 95%CI=1.27-1.44). Reduced ORs of road rule violations (OR=0.66, 95%CI=0.62-0.70) and wearing a helmet (OR=0.39, 95%CI=0.32-0.49) were found to be associated with riding pedal-equipped E-bikes compared with those in scooter form; whereas the highest elevated ORs of Helmet use (OR=7.21,95%CI=4.01-12.98) and road rule violations (OR=5.34, 95%CI=3.58-7.99) were observed among couriers compared with other E-bikers [Table 3].

For peer review only

Table 3. Adjusted Odds Ratios (95% Confidence Intervals) for speeding, road rule violations, and helmet use among E-bikers *

		Speeding N=800	Violations N=20,647	Helmet use N=20,647
Region	Urban	1.14 (0.66-1.99)	1.01 (0.95-1.07)	0.98 (0.84-1.14)
	Rural		reference	
Weather	Sunny	0.29 (0.02-3.58)	0.89 (0.40-1.98)	0.58 (0.19-1. 80)
	Cloudy	--	0.76 (0.31-1.88)	0.55 (0.16-1.98)
	Rainy		reference	
Weekday	Yes	1.73 (0.92-3.25)	0.86 (0.53-1.42)	1.43 (0.72-2.81)
	No		reference	
Time of day	Morning	0.73 (0.47-1.15)	1.07 (0.69-1.65)	1.01 (0.56-1.82)
	Afternoon		reference	
Volume	Basic	0.27 (0.02-3.24)	1.53 (0.77-3.02)	0.86 (0.35-2.14)
	Low	0.50 (0.04-6.11)	1.29 (0.62-2.67)	0.90 (0.34-2.37)
	Medium	0.16 (0.01-2.06)	1.13 (0.36-3.60)	0.66 (0.14-3.02)
	High		reference	
Traffic control	Yes	--	0.76 (0.41-1.38)	1.57 (0.70-3.51)
	No		reference	
Gender	Male	**2.12 (1.50-3.01)**	**1.35 (1.27-1.44)**	0.66 (0.54-0.80)
	Female		reference	
Courier	Yes	0.75 (0.20-2.82)	**5.34 (3.58-7.99)**	**7.21 (4.01-12.98)**
	No		reference	
Registration	Yes	0.96 (0.63-1.44)	**0.82 (0.75-0.88)**	1.18 (0.92-1.52)
	No		reference	
Pedals	Yes	0.79 (0.56-1.12)	**0.66 (0.62-0.70)**	**0.39 (0.32-0.49)**
	No		reference	

* The adjusting variables were observational items showed in Table1.
Note: Significant results are highlighted in bold

DISCUSSION

Poor safety practice was commonplace including speeding, road rule violations, and little use of helmets and this did not vary between rural and urban areas. Male E-bikers seemed to bear more risks of speeding and road rule violations. Although couriers were 7 times more likely to wear a helmet when riding an E-bike, they were also 5 times more likely to violate road rules when entering an intersection compared with the other E-bikers. When riding E-bikes with pedals rather than those in scooter form, E-bikers had a lower likelihood of violating road rules and wearing a helmet. These identified safety gaps build on previous evidence[5-10] identifying the need to discourage unsafe practice and encourage safety gear use among

For peer review only - http://bmjopen.bmj.com/site/about/guidelines.xhtml

E-bikers in China, particularly in the context of China recently joining global action to improve road safety in the next decade.[11]

Consistent with previous studies,[2, 8-10] this study confirmed a range of factors associated with observed E-biker behaviours and revealed the invariant nature of unsafe E-bike riding practice in general. We conducted a similar study during March 2012 in metropolitan Suzhou areas and found 27% of E-bikers violated at least one road rule and 41% used at least one type of safety gear.[10] The current study identified a somewhat higher prevalence of road rule violations (38%) and lower safety gear use (3%). This variation may be explained by the seasonality, e.g., the sharp drop in glove use (from 37% to 0.6%) and helmet use (from 9% to 2%). Zhang et al reported a similar decrease in helmet use among motorcycles in Guangxi during the hot and humid season.[14] The study findings also relate to the previous reports of increasing E-biker fatalities and injuries across mainland China,[4-7] which echoes the call for action to develop policies to improve E-bike safety in China.

The observed high prevalence of unsafe riding practices implies a need for policy change. Current road rules regulate E-bikes as pedal bicycles that should travel in non-motor vehicle lanes at a maximum speed of 15 km/h,[15] whereas the mandatory Standard for E-bikes specifies a maximum speed of 20 kilometers per hour and a maximum weight of 40 kilograms in addition to requiring a specified braking distance and pedal installment.[13]

However, these specifications may not be widely enforced as most of the electric two-wheelers are not designed and produced in line with the national standards of non-motor vehicles.[16] With regard to those that do comply with the national standards of non-motor vehicles, producers, for marketing purposes, often install the so called "speed limiting devices" on their products. With the speed limiting devices, the maximum speed by which the electric two-wheelers could operate is 20 Km/h as required by the mandatory Standard for E-bikes; whereas, the speed limiting devices are designed and installed in a way that could be easily dismantled by customers themselves or sales persons. Without speed limiting devices,

For peer review only - http://bmjopen.bmj.com/site/about/guidelines.xhtml

the speed of these electric two-wheelers could effortlessly go beyond 20 Km/h and maybe up to 40 Km/h [17].

Notably, the *Safety Specifications for Power Driven Vehicles Operating on Roads* defines a motorcycle as being power-driven with the maximum speed exceeding 50 kilometers per hour and a moped with a maximum speed range from 20-50 kilometers per hour.[18] This has the legal implication that any E-bikes (mostly in scooter form) that could travel faster than 20 kilometers per hour should be regulated as motor vehicles by the road rules. Obviously, such conflict between the mandatory Standard for E-bikes and road rules might create difficulties for legislative enforcement, including cities where motorcycles are banned.

In addition, the effectiveness of helmets in head injury prevention is well established for both bicyclists [19] and motorcyclists.[20] Regardless of the introduction of compulsory motorcycle helmet use producing a substantial increase in use of helmets among motorcyclists in China,[21] similar regulations were missing for bicyclists and E-bikers. Therefore, road rule revisions to encourage helmet use among E-bikers are urgently needed and should be incorporated into the broad road safety agenda.

To our best knowledge, no international E-bikers` riding practice studies were conducted before. Compared with other cross-sectional observational studies, this study is limited by lack of generalizability to other settings (different regions); possible bias due to unmeasurable confounding (influence of road infrastructure); and possible misclassification due to measurement errors (incorrect speed reading). Thus, care should be taken when interpreting the study findings. To minimize the likelihood of measurement errors, various small-scale pilot studies were conducted to determine the feasibility of the study and to validate the observational instruments. Moreover, this study established a strict quality control scheme and recruited experienced observers who had participated in previous studies using similar techniques.[10] Thus, misclassification may not bias the key findings to an important degree.

For peer review only - http://bmjopen.bmj.com/site/about/guidelines.xhtml

Nevertheless, the study findings provide new evidence to complement previous findings as to diverse safety issues among E-bikers, and to reinforce the imperative to encourage safety gear use and discourage unsafe on-road practices.

CONCLUSION

E-bikes are becoming a dominant road transportation means for commuters in China, and they are increasingly used as a sustainable alternative to traditional transportation in other countries because of the low maintenance cost and low polluting mobility. The observed unsafe riding practices signal emerging road safety challenges in China and in similar settings elsewhere. Translating established safety practices such as helmet use and enforcing existing countermeasures such as speed limit devices may be used to improve safety practice among E-bikers. Strong political will is especially needed to leapfrog substantial losses associated with E-bike risk in China without sacrificing mobility needs.

ACKNOWLEDGEMENT

This project has funding support from Bloomberg Philanthropies as part of the Bloomberg Philanthropies Global Road Safety Programme in China. Additional funding was provided by Jiangsu Provincial Centre for Disease Control and Prevention (JKRC2011014). We appreciate special support from the WHO China Office, the Ministry of Health in China, Suzhou Bureau of Health, and Suzhou Bureau of Public Security. We also thank Ziyi Jin, Jianfeng Liu, Qi Zhang, Xianglin Liu, and Yan Lu for their assistance in field work. Wei Du is supported by an NHMRC fellowship. Yilan Liao is supported by the National Natural Science Foundation of China (No. 41101431）.

CONTRIBUTION

Jie Yang and Yihe Hu contributed equally to the study design, research implementation, literature review, data analysis, writing full first draft and contributed to subsequent drafts. All the other authors contributed to the conceptual development, data interpretation, critical

For peer review only - http://bmjopen.bmj.com/site/about/guidelines.xhtml

revision of the first manuscript, and subsequent drafts.

COMPETING INTERESTS: There is no conflict of interest.

REFERENCES

1 Weinert J, Ma C, Cherry C. The transition to electric bikes in China: history and key reasons for rapid growth. Transportation 2007;34: 301-318

2 2Yao L, Wu C. Traffic safety of e-bike riders in China: safety attitudes, risk perception and aberrant riding behaviors. Transportation Research Record: Journal of the Transportation Research Board & Transportation Research Board 91st Annual Meeting 2012: Paper 4599.

3 3Hurst D, Wheelock C. Electric two-wheel vehicles (electric bicycles, mopeds, scooters, and motorcycles): market analysis and forecasts. Boulder, Colorado: Pike Research, 2010.

4 Bureau of Traffic Management (2004-2010). China road traffic accidents statistics, 2004 to 2010. Beijing: Ministry of Public Security.

5 Du W, Yang J, Powis B, et al. Epidemiological profile of hospitalised injuries among electric bicycle riders admitted to a rural hospital in Suzhou: a cross sectional study. Injury Prevention 2013 Jun 1. [Epub ahead of print].

6 Duan L, Wu C, Deng X, et al. Road traffic injuries in China, 2006–2008. Chinese Journal of Public Health and Preventive Medicine 2010;21:10-13.

7 Feng Z, Raghuwanshi R, Xu Z, et al. Electric-bicycle-related injury: a rising traffic injury burden in China. Injury Prevention 2010;16: 417-419.

8 Weinert J, Ma C, Yang X, et al. Electric Two-Wheelers in China: Effect on Travel Behavior, Mode Shift, and User Safety Perceptions in a Medium-Sized City. Transportation Research Record: Journal of the Transportation Research Board 2007; 2038:62-68.

9 Wu C, Yao L, Zhang K. The red-light running behavior of electric bike riders and cyclists at urban intersections in China: An observational study. Accident Analysis & Prevention, Available online 2011 July 2.

10 Du W, Yang J, Powis B, et al. Understanding on-road practices of electric bike riders: an observational study in a developed city of China. Accident Analysis and Prevention 2013 Jun 25;59C:319-326. [Epub ahead of print]

11 World Health Organization. Global plan for the decade of action for road safety 2011-2020. Geneva: WHO, 2011. Available from:http://www.who.int/roadsafety/decade_of_action/plan/plan_english.pdf (accessed 10 Aug 2013)

12 http://www.bushnell.com/all-products/outdoor-technology/velocity-speed-gun. (accessed 1 Nov 2013)

13 Standardization Administration of the People’s Republic of China (1999). General technical

For peer review only - http://bmjopen.bmj.com/site/about/guidelines.xhtml

standards of Electric bicycles (GB17761-1999). Chinese Standards Publisher, 1999.

14 Zhang J, Norton R, Tang K, et al. Motorcycle ownership and injury in China. Int. J. Injury Control and Safety Promotion 2004;11:159-163.

15 China National People's Congress. Law of the People's Republic of China on road traffic safety. Beijing, 2004.

16 Tsinghua Law School. Aiming at Behavioral Changes: Improving Drink Driving and Speeding Law in China Beijing: Tsinghua University, 2012 (Unpublished Report).

17 http://njrb.njnews.cn/html/2011-03/14/node_69.htm. (accessed 10 August 2013).

18 Standardization Administration of the People's Republic of China. Safety Specifications for Power Driven Vehicles Operating on Roads (GB7258-2004). Chinese Standards Publisher, 2004.

19 Thompson D, Rivara F, Thompson R. Helmets for preventing head and facial injuries in bicyclists. Cochrane Database of Systematic Reviews (published in Issue 4, 1999).

20 Liu B, Ivers R, Norton R,et al. Helmets for preventing injury in motorcycle riders. Cochrane Database of Systematic Reviews,(published in Issue 1, 2008).

21 Yu J, Ke L, Ivers R, Du W, Senserrick T. Prevalence rates of helmet use among motorcycle riders in a developed region in China. Accident Analysis and Prevention 2011;43:214-219.

For peer review only

STROBE 2007 (v4) Statement—Checklist of items that should be included in reports of *cross-sectional studies*

Section/Topic	Item #	Recommendation	Reported on page #
**Title and abstract**	1	(*a*) Indicate the study's design with a commonly used term in the title or the abstract	2
		(*b*) Provide in the abstract an informative and balanced summary of what was done and what was found	2
**Introduction**			
Background/rationale	2	Explain the scientific background and rationale for the investigation being reported	4
Objectives	3	State specific objectives, including any prespecified hypotheses	4
**Methods**			
Study design	4	Present key elements of study design early in the paper	5
Setting	5	Describe the setting, locations, and relevant dates, including periods of recruitment, exposure, follow-up, and data collection	5
Participants	6	(*a*) Give the eligibility criteria, and the sources and methods of selection of participants	5,6
Variables	7	Clearly define all outcomes, exposures, predictors, potential confounders, and effect modifiers. Give diagnostic criteria, if applicable	7
Data sources/ measurement	8*	For each variable of interest, give sources of data and details of methods of assessment (measurement). Describe comparability of assessment methods if there is more than one group	6,7
Bias	9	Describe any efforts to address potential sources of bias	*13*
Study size	10	Explain how the study size was arrived at	7
Quantitative variables	11	Explain how quantitative variables were handled in the analyses. If applicable, describe which groupings were chosen and why	6,7
Statistical methods	12	(*a*) Describe all statistical methods, including those used to control for confounding	8
		(*b*) Describe any methods used to examine subgroups and interactions	8
		(*c*) Explain how missing data were addressed	Not applicable
		(*d*) If applicable, describe analytical methods taking account of sampling strategy	Not applicable
		(*e*) Describe any sensitivity analyses	Not applicable
**Results**			

For peer review only

Participants	13*	(a) Report numbers of individuals at each stage of study—eg numbers potentially eligible, examined for eligibility, confirmed eligible, included in the study, completing follow-up, and analysed	8
		(b) Give reasons for non-participation at each stage	Not applicable
		(c) Consider use of a flow diagram	Not applicable
Descriptive data	14*	(a) Give characteristics of study participants (eg demographic, clinical, social) and information on exposures and potential confounders	8,9
		(b) Indicate number of participants with missing data for each variable of interest	-
Outcome data	15*	Report numbers of outcome events or summary measures	8,9
Main results	16	(*a*) Give unadjusted estimates and, if applicable, confounder-adjusted estimates and their precision (eg, 95% confidence interval). Make clear which confounders were adjusted for and why they were included	10
		(*b*) Report category boundaries when continuous variables were categorized	Yes
		(*c*) If relevant, consider translating estimates of relative risk into absolute risk for a meaningful time period	Not applicable
Other analyses	17	Report other analyses done—eg analyses of subgroups and interactions, and sensitivity analyses	Not applicable
**Discussion**			
Key results	18	Summarise key results with reference to study objectives	11
Limitations	19	Discuss limitations of the study, taking into account sources of potential bias or imprecision. Discuss both direction and magnitude of any potential bias	13
Interpretation	20	Give a cautious overall interpretation of results considering objectives, limitations, multiplicity of analyses, results from similar studies, and other relevant evidence	13
Generalisability	21	Discuss the generalisability (external validity) of the study results	14
**Other information**			
Funding	22	Give the source of funding and the role of the funders for the present study and, if applicable, for the original study on which the present article is based	14

*Give information separately for cases and controls in case-control studies and, if applicable, for exposed and unexposed groups in cohort and cross-sectional studies.

Note: An Explanation and Elaboration article discusses each checklist item and gives methodological background and published examples of transparent reporting. The STROBE checklist is best used in conjunction with this article (freely available on the Web sites of PLoS Medicine at http://www.plosmedicine.org/, Annals of Internal Medicine at http://www.annals.org/, and Epidemiology at http://www.epidem.com/). Information on the STROBE Initiative is available at www.strobe-statement.org.

For peer review only

Unsafe riding practice among electric bikers in Suzhou, China: an observational study

Journal:	*BMJ Open*
Manuscript ID:	bmjopen-2013-003902.R2
Article Type:	Research
Date Submitted by the Author:	19-Nov-2013
Complete List of Authors:	Yang, Jie; Jiangsu provincial centre for disease prevention and control, NCD prevention<br>Hu, Yihe; Suzhou municipal centre for disease prevention and control, NCD prevention<br>Du, Wei; Peking University, Institute of Population Research; University of New South Wales, Neuroscience Research Australia<br>Powis, Brent; International Medical University, School of Postgraduate Studies and Research<br>Ozanne-Smith, Joan; Monash University, Department of Forensic Medicine<br>Liao, Yilan; Chinese Academy of Sciences, Institute of Geographical Sciences and Nature Resources Research<br>Li, Ning; Peking University, Institute of Population Research<br>Wu, Ming; Jiangsu provincial centre for disease prevention and control, NCD prevention
**Primary Subject Heading**:	Epidemiology
Secondary Subject Heading:	Public health, Epidemiology
Keywords:	Electric Bike, Cross Sectional Study, Behavior, Risk Factor Research, Driver

SCHOLARONE™ Manuscripts

For peer review only - http://bmjopen.bmj.com/site/about/guidelines.xhtml

Title

Unsafe riding practice among electric bikers in Suzhou, China: an observational study

Corresponding Author

Dr. Ming Wu

Tel/Fax: +86 (25) 8375 9411

Email: wuming@jscdc.cn

Address: Department of Non-communicated Chronic Disease Prevention and Control, Jiangsu Provincial Center for Disease Prevention and Control, Nanjing 210009, China

Authors

Jie Yang, MSc[1*], Yihe Hu, MBBS[2*], Wei Du, PhD[3,4], Brent Powis, PhD[5], Joan Ozanne-Smith, MD[6], Yilan Liao, PhD[7], Ning Li, PhD[3], Ming Wu, PhD[1]

1. Department of Chronic Disease Control, Jiangsu Provincial Centre for Disease Control and Prevention, China
2. Department of Chronic Disease Control, Suzhou Municipal Centre for Disease Control and Prevention, Suzhou, China
3. Institute of Population Research, Peking University, China
4. Neuroscience Research Australia, University of New South Wales, Australia
5. School of Postgraduate Studies and Research, International Medical University, Malaysia
6. Department of Forensic Medicine, Monash University, Australia
7. Institute of Geographical Sciences and Nature Resources Research, Chinese Academy of Sciences, China

* These two authors contributed equally

Key Words: Electric Bike, Cross Sectional Study, Behavior, Risk Factor Research, Driver.

Word count: 2,705 words

For peer review only

For peer review only - http://bmjopen.bmj.com/site/about/guidelines.xhtml

Abstract

Background: Increasing Electric bike (E-bike) related fatalities have been increasing rapidly in China and such injuries may be partly attributable to unsafe riding practice.

Objectives: To describe potentially unsafe riding behaviors among E-bikers and to investigate factors influencing these practices in China.

Methods: In September 2012, a cross-sectional observation study including a speed measurement component was conducted in Wuzhong (an urban District) and Zhangjiagang (a rural District) of Suzhou, Jiangsu Province, China. Hand held radar speed meters were used to read traveling speeds of E-bikes and a pro-forma observation checklist was used to collect data on road riding practice. Mixed-effect logistic regressions were used to calculate adjusted Odds Ratios (ORs) and 95% Confidence Intervals (CIs) for the association between speeding, road rule violations and helmet use and their influencing factors.

Results: Among 800 E-bikes with a speed reading, 70.9% exceeded the designed speed limit of 20 km/h. Among a further 20,647 E-bikers observed, 38.3% did not comply with the road rules when entering intersections; and only 2.2% wore helmets. No regional variation was identified between urban and rural areas. Male was associated with more speeding and road rule violations; whereas riding a pedal-equipped E-bike was associated with less road rule violations and less helmet use.

Conclusion: Unsafe riding practices such as speeding, road rule violations, and lack of helmet use were commonplace among E-bikers, especially among males. The study findings indicate that measures aimed at improving e-bike safety are required in China.

For peer review only

Article Summary

Article Focus:

- E-biker safety is an emerging public health challenge in China. This study focuses on the unsafe on-road riding behaviours among Chinese E-bikers and factors influencing these practices.

Key Message:

- Direct roadside observation techniques were applied to describe safety practices of E-bikers and hand held radar meters were used to estimate their actual travelling speed.
- The variation of on-road riding behaviours between rural and urban areas was evaluated.
- Factors influencing observed riding behaviours were further investigated.

Strengths and Limitations of the Study:

- In this study, we evaluated how fast E-bikers ride on roads and the possible regional variation of riding behaviours. Furthermore, we investigated factors influencing observed riding behaviours.
- Study findings could provide new evidence to enhance the understanding of on-road riding behaviours among Chinese E-bikers, and to reinforce the imperative to encourage safety gear use and discourage unsafe on-road practices.
- Findings might be limited by lack of generalizability to other settings, possible bias due to unmeasurable confounding, and possible misclassification due to measurement errors.

For peer review only

INTRODUCTION

In China, the last few years have witnessed the rapid growth of E-bikes (either with pedals or in scooter form) due to increasing mobility demand when public transportation systems are crowded and inconveniently routed.[1] Growing wealth among Chinese also increases affordability of E-bike purchase, normally priced at approximately USD 300. In China, more than 120 million E-bikes were registered by 2011 [2] and globally an estimated 466 million E-bikes are expected to hit the road by 2016. [3]

In China, bicycle use is shifting to E-bike use. Unfortunately, an associated unwanted shift was observed as E-bike related fatalities increased almost 7 times over time from 589 in 2004 to 4,029 in 2010 across the nation, whereas bicycle related fatalities decreased 3 times approximately from 13,655 to 4616 during the same period. [4] Moreover, E-bikers hospitalized for injuries accounted for 57% of serious non-fatal road traffic injuries and 50% of the direct hospitalization cost for all road crash casualties in a rural hospital in Suzhou.[5] Thus, E-biker safety is an emerging public health challenge in China .[5-7]

Whilst unsafe riding practices have been reported among E-bikers using a self-reported survey,[2,8] synchronized video camera recording techniques,[9] and direct roadside observations,[10] no studies have reported on how fast E-bikers ride on roads allowing for possible regional variation, such as rural/urban disparities, commonly observed for other road safety issues. To enhance the understanding of on-road riding behaviors among Chinese E-bikers including evaluation of rural/urban variation, we used direct roadside observation techniques to describe their safety practices and hand held radar meters to estimate their actual traveling speed. We further investigated factors influencing these observed behaviors.

METHODS

We applied a cross-sectional observation research which comprised two components for this study, i.e., observations *with* or *without* speed measurement, which were conducted separately in Suzhou, one of the intervention pilot cities in China for the Bloomberg

For peer review only - http://bmjopen.bmj.com/site/about/guidelines.xhtml

Philanthropies Global Road Safety Programme (A multinational programme which take effort to reduce death and serious injury on the roads in ten low- and middle-income countries over five years extending from 2010 to 2014). [11] Suzhou has the sixth highest gross domestic product (GDP) per capita on the Chinese mainland, a resident population of 10 million and at least 2 million E-bikes. [10] The study protocol was approved by the Ethics Committee of Jiangsu Provincial Centre for Disease Control and Prevention.

Field implementation

In Suzhou, two administrative districts, i.e., Wuzhong (urban district) and Zhangjiagang (rural district) were selected to conduct both study components. Wuzhong is located in the south of Suzhou metropolitan regions with a resident population of 606,231 in 2012, comprising 49.0% males and 51.0% females; whereas Zhangjiagang is located to the north of Suzhou metropolitan regions with a resident population of 909,038 in 2012, comprising 49.2% males and 50.8% females. To select observation sites, a grid was placed over standard maps of Wuzhong and Zhangjiagang, random digits were generated for each grid box for selection and each valid grid box contained at least one intersection having traffic lights. For each randomly selected site, an alternate site was also selected randomly from the grid as a backup. A pilot study was carried out to validate the field feasibility such as having low volumes of E-bikes for speed measurement; at least two-way motor vehicle lanes, pedestrian crossings, and bicycle lanes; enough distance between observation sites so the same E-bikers were unlikely to be observed twice; less likely to interrupt observed behaviors and least likely to increase the crash risk for observers. A total of eight sites (i.e, two from each district for each study component) were randomly selected.

For the study component with speed measurement, observers concealed themselves at approximately 50 meters from the corner of the intersection, used handheld radar speed meters (Bushnell Velocity 10-1911CM with measurement range of 16-320km/h) [12] to record the speed meter reading, and collected information on on-road riding behaviors among oncoming E-bikers with valid speed meter reading. For the study component without speed

For peer review only - http://bmjopen.bmj.com/site/about/guidelines.xhtml

measurement, the observations were conducted at intersection corners. Considering traffic characteristics may vary every day, we randomly selected 4 days in a week including one weekend day (September 11th, 12th, 13th, and 15th) for the study component with speed measurement and selected another 7-day period (September 17th-23th) for the study component without speed measurement. The time of day (7.00am–6.59pm) for site observations was set at 2-hour intervals as observational periods and randomly assigned to sites.

Four experienced observers were recruited, who had participated in previous roadside E-bike observation studies in other districts in Suzhou.[10] Prior to field implementation, the site observers were trained in specifications of different behaviors, identification of different types of protective items, techniques to observe multiple behaviors especially when an E-bike was moving, and data quality control. The site observers formed two groups, i.e., the urban group and rural group without rotation. Each group remained in the same district and changed their observational periods and sites every day. Roadside pilot observations and regular on-site audits were conducted to ensure the safety guidelines and accurate and appropriate implementation of the data collection process.

The observers worked in pairs, to observe oncoming E-bikes in ascending distance order. Data items were collected on a pro-forma checklist including type of E-bike (with bike pedals or in scooter form), registration status, rider's gender, couriers or not (In China, many couriers are required to wear uniforms when working and companies provide them uniforms with own logos; although uniforms are in different styles, it's easy to differentiate couriers from normal E-bikers), carrying passengers, carrying oversized cargo (estimated >60 x 40 x 20cm^3, the size of normal airlines carry-on luggage), riding in a motor vehicle lane, running red lights, riding in the opposite direction (i.e., facing oncoming traffic), using mobile phone, using helmet, wearing leather gloves, and wearing other motorcycle protective clothing. Weather, day of week, time of day, average E-bike traffic volume per minute, presence of a traffic controller (traffic policeman or traffic police assistant directing vehicular and pedestrian traffic) were recorded on separate data collection forms.[Table 1] Regarding the speed measurement

For peer review only - http://bmjopen.bmj.com/site/about/guidelines.xhtml

component, for every tenth E-bike, the second observer recorded the radar speed reading during the speed measurement observations.

Outcome of interest

1) Speeding was defined as binary, i.e., yes (travel speed >20 km/h) or no (travel speed ≤20km/h), because E-bikes are manufactured to a mandatory Standard [13] with designed maximum travel speed of 20 km/h;
2) Violation was defined as binary, i.e., yes (at least one of the following road rule violations was observed: carrying passengers, carrying oversized cargo, riding in a motor vehicle lane, running red lights, riding in the opposite direction or using mobile phone), or no;
3) Helmet use was defined as binary, i.e., yes (wearing a motorcycle helmet) or no.

Table 1. Observational item categorization

Observational items	Categorization
weather	sunny, cloudy, or rainy
day of week	weekday or weekend
time of day	morning or afternoon
average E-bike traffic volume per minute	basic (≤10 E-bikes), low (11-15 E-bikes), medium (16-20 E-bikes), or high (>20 E-bikes)
presence of a traffic controller (traffic policeman or traffic police assistant directing vehicular and pedestrian traffic)	yes or no
type of E-bike	equipped with pedals or otherwise in scooter form
E-bike registration status	registered with registration plate displayed or otherwise unregistered;
E-bikers' gender	male or female
E-bikers' occupation	couriers or not
carrying passengers	yes or no
carrying oversized cargo (>60 x 40 x 20cm^3)	yes or no
riding in a motor vehicle lane	yes or no
running red lights	yes or no
riding in the opposite direction (i.e., facing oncoming traffic)	yes or no
mobile phone use	yes or no
helmet use	yes or no
wearing leather gloves	yes or no
wearing other motorcycle protective clothing	yes or no

Statistical analysis

Completed observational records were reviewed; and data were entered with double entry. All

For peer review only - http://bmjopen.bmj.com/site/about/guidelines.xhtml

data analyses were conducted using SAS version 9.2 (SAS Institute, 2002). The inter-observer reliability was assessed using Kappa statistics and agreement reached at least 85% for each pair of observers. Frequencies and proportions of speed reading and observed on-road riding behaviors were calculated where appropriate. We used mixed logistic regression allowing for random selection of observational sites to estimate Odds Ratio (OR) and associated 95% Confidence Interval (CI) for different study outcomes adjusted for observational items. [Table 1] Further mixed-effect logistic regression analyses of individual road rule violations (i.e., carrying passengers, carrying oversized cargo, riding in a motor vehicle lane, running red lights, riding in the opposite direction or using mobile phone) were stratified by different regions, i.e., Wuzhong or Zhangjiagang. We defined P-values less than 0.05 as statistically significant.

For peer review only

RESULTS

There were a total of 27 observational periods (i.e., 14 in Wuzhong and 13 in Zhangjiagang) for direct observational data collection on 20,647 E-bikes, and 16 periods (i.e., 8 for each district) for speed measurement on 800 E-bikes. The average number of E-bikes per observational period was 729 (range: 103 to 1317) and 803 (range: 552 to 1046) for Wuzhong and Zhangjiagang, respectively.

Table 2 Distribution of observational items among E-bike study populations

		Wuzhong (Urban district)		Zhangjiagang (Rural district)	
		with speed measure	without speed measure	with speed measure	without speed measure
Sample size	(n)	400	10202	400	10445
Traffic mix	E-bikes (%)	44.6	46.5	28.2	34.5
	Pedestrians (%)	7.4	9.1	3.0	10.6
	Bicycles (%)	3.5	3.2	9.6	4.7
	Cars (%)	36.2	35.1	57.1	47.6
	Heavy vehicles （%）	8.3	6.2	2.2	2.7
E-bike volume per minute (basic as ≤10 E-bikes, low as 11-15 E-bikes, medium as16-20 E-bikes, or high as >20 E-bikes)	Basic (%)	25.0	40.9	50.0	40.2
	Low (%)	12.5	17.0	37.5	59.8
	Medium (%)	37.5	10.4	12.5	0.0
	High (%)	25.0	31.7	0.0	0.0

For peer review only - http://bmjopen.bmj.com/site/about/guidelines.xhtml

Weather	Sunny (%)	75.0	82.8	100.0	69.8
	Cloudy (%)	0.0	9.4	0.0	30.2
	Rainy (%)	25.0	7.8	0.0	0.0
Day of week	Weekday (%)	75.0	75.7	75.0	70.5
Time of day	Morning (%)	37.5	45.7	50.0	39.5
Traffic controller	Yes (%)	Not applicable	0.0	Not applicable	25.4
Occupation	Courier (%)	1.8	0.8	1.3	0.5
Gender	Males (%)	69.0	59.8	58.5	51.7
Registration	Yes (%)	94.0	96.3	35.3	36.3
Pedals	Yes (%)	28.0	37.1	53.3	54.3
Carrying passengers	Yes (%)	24.3	21.3	11.0	20.5
Carrying large cargo	Yes (%)	11.5	6.9	15.8	10.1
Riding in a motor vehicle lane	Yes (%)	13.5	3.1	2.3	1.4
Riding opposite direction	Yes (%)	3.0	5.7	25.0	30.8
Mobile phone use	Yes (%)	0.8	0.7	2.0	1.1
Helmet use	Yes (%)	3.3	2.1	5.0	2.2
Gloves	Yes (%)	0.8	0.4	1.8	0.8
Running red lights	Yes (%)	Not applicable	16.5	Not applicable	6.2

Table 2 describes the observation results and shows that E-bikes were the dominant transportation means in Wuzhong district. Despite similarities across some observational items such as low helmet use (i.e., 2.1% in Wuzhong vs 2.2% in Zhangjiagang) and commonplace carrying of passengers (21.3% vs 20.5%), there were differences in riding violations especially for riding licensed E-bikes (96.3% vs 36.3%) [Table 2].

Approximately 83.3% (n=333) and 58.5% (n=234) E-bikers were observed traveling at a speed greater than 20 km/h; approximately 41.3% (n=4,211) and 35.4% (n=3,700) violating at least one of the listed road rules; and 2.5% (n=251) and 3.1% (n=319) using any safety gear in Wuzhong and Zhangjiagang, respectively.

No statistically significant evidence indicates the existence of regional variation in terms of elevated odds of speeding, general road rule violations, or lack of helmet use. Compared with female E-bikers, males showed greater ORs of speeding (OR=2.12, 95%CI=1.50-3.01) and violation (OR=1.35, 95%CI=1.27-1.44). Reduced ORs of road rule violations (OR=0.66, 95%CI=0.62-0.70) and wearing a helmet (OR=0.39, 95%CI=0.32-0.49) were found to be associated with riding pedal-equipped E-bikes compared with those in scooter form; whereas the highest elevated ORs of Helmet use (OR=7.21,95%CI=4.01-12.98) and road rule

For peer review only - http://bmjopen.bmj.com/site/about/guidelines.xhtml

violations (OR=5.34, 95%CI=3.58-7.99) were observed among couriers compared with other E-bikers [Table 3].

Table 3. Adjusted Odds Ratios (95% Confidence Intervals) for speeding, road rule violations, and helmet use among E-bikers *

		Speeding N=800	Violations N=20,647	Helmet use N=20,647
Region	Urban	1.14 (0.66-1.99)	1.01 (0.95-1.07)	0.98 (0.84-1.14)
	Rural		reference	
Weather	Sunny	0.29 (0.02-3.58)	0.89 (0.40-1.98)	0.58 (0.19-1. 80)
	Cloudy	--	0.76 (0.31-1.88)	0.55 (0.16-1.98)
	Rainy		reference	
Weekday	Yes	1.73 (0.92-3.25)	0.86 (0.53-1.42)	1.43 (0.72-2.81)
	No		reference	
Time of day	Morning	0.73 (0.47-1.15)	1.07 (0.69-1.65)	1.01 (0.56-1.82)
	Afternoon		reference	
Volume	Basic	0.27 (0.02-3.24)	1.53 (0.77-3.02)	0.86 (0.35-2.14)
	Low	0.50 (0.04-6.11)	1.29 (0.62-2.67)	0.90 (0.34-2.37)
	Medium	0.16 (0.01-2.06)	1.13 (0.36-3.60)	0.66 (0.14-3.02)
	High		reference	
Traffic control	Yes	--	0.76 (0.41-1.38)	1.57 (0.70-3.51)
	No		reference	
Gender	Male	**2.12 (1.50-3.01)**	**1.35 (1.27-1.44)**	0.66 (0.54-0.80)
	Female		reference	
Courier	Yes	0.75 (0.20-2.82)	**5.34 (3.58-7.99)**	**7.21 (4.01-12.98)**
	No		reference	
Registration	Yes	0.96 (0.63-1.44)	**0.82 (0.75-0.88)**	1.18 (0.92-1.52)
	No		reference	
Pedals	Yes	0.79 (0.56-1.12)	**0.66 (0.62-0.70)**	**0.39 (0.32-0.49)**
	No		reference	

* The adjusting variables were observational items showed in Table1.
Note: Significant results are highlighted in bold

The results of regional stratification demonstrate that risk factor profile may vary across regions. For example of riding opposite direction, couriers were associated with significantly elevated OR (2.03, 95%CI=1.03-4.00) in Wuzhong but marginally reduced OR (0.71, 95%CI=0.36-1.41) in Zhangjiagang; whereas males were associated with marginally elevated (1.09, 95%CI=0.91-1.31) in Wuzhong but significantly reduced OR (0.89, 95%CI=0.81-0.97) in Zhangjiagang [Tables 4 and 5].

For peer review only - http://bmjopen.bmj.com/site/about/guidelines.xhtml

Table 4. Adjusted Odds Ratios (95% Confidence Intervals) for individual road rule violations among E-bikers observed in Wuzhong (Urban districtu)*

		Carrying passengers N=2,169	Running red lights N=1,682	Carrying large cargo N=700	Riding opposite direction N=579	Riding in a motor vehicle lane N=315	Mobile phone use N=72
Weather	Sunny	2.51 (0.64-9.86)	0.41 (0.06-2.58)	1.16 (0.62-2.18)	1.54 (0.49-4.84)	1.03 (0.07-15.00)	0.80 (0.16-4.05)
	Cloudy	2.01 (0.32-12.74)	0.78 (0.07-9.51)	1.28 (0.56-2.93)	1.43 (0.31-6.62)	7.72 (0.21-288.20)	3.17 (0.34-29.69)
	Rainy			reference			
Weekday	Yes	0.55 (0.16-1.92)	1.21 (0.22-6.62)	1.15 (0.64-2.06)	0.83 (0.29-2.36)	0.45 (0.04-5.31)	0.98 (0.14-6.69)
	No			reference			
Time of day	Morning	0.71 (0.22-2.33)	2.35 (0.47-11.70)	1.22 (0.70-2.10)	0.90 (0.34-2.42)	0.86 (0.08-8.97)	1.60 (0.32-8.00)
	Afternoon			reference			
Volume	Basic	0.53 (0.15-1.92)	9.21 (1.63-51.92)	**3.59 (2.01-6.41)**	0.90 (0.31-2.59)	**47.54 (3.69-612.34)**	5.15 (1.13-23.42)
	Low	0.65 (0.11-4.01)	4.95 (0.43-57.61)	**2.65 (1.12-6.25)**	0.86 (0.19-3.98)	3.86 (0.11-133.67)	3.51 (0.32-37.89)
	Medium	0.43 (0.07-2.76)	8.41 (0.68-104.11)	**1.88 (0.81-4.36)**	0.83 (0.18-3.89)	7.59 (0.20-296.13)	4.67 (0.43-50.91)
	High			reference			
Gender	Male	**1.26 (1.13-1.40)**	**1.32 (1.17-1.49)**	**1.73 (1.43-2.10)**	1.09 (0.91-1.31)	**2.14 (1.62-2.84)**	**2.38 (1.29-4.40)**
	Female			reference			
Courier	Yes	**0.11 (0.03-0.43)**	0.82 (0.44-1.55)	**53.36 31.34-90.86)**	**2.03 (1.03-4.00)**	1.14 (0.34-3.76)	**5.26 (1.79-15.46)**
	No			reference			
Registrations	Yes	1.17 (0.89-1.54)	0.99 (0.75-1.32)	**0.69 (0.48-0.99)**	1.07 (0.68-1.66)	0.74 (0.45-1.23)	3.16 (0.43-22.94)
	No			reference			
Pedals	Yes	**0.69 (0.62-0.77)**	0.89 (0.79-1.01)	**0.62 (0.51-0.75)**	0.99 (0.83-1.20)	**0.57 (0.43-0.77)**	0.82 (0.47-1.42)
	No			reference			

* Odds Ratios were adjusted for observational items in Table 1 (significant results are highlighted in bold).

For peer review only - http://bmjopen.bmj.com/site/about/guidelines.xhtml

Table 5. Adjusted Odds Ratios (95% Confidence Intervals) for individual road rule violations among E-bikers observed in Zhangjiagang (Rural district)*

		Riding opposite direction N=3,220	Carrying passengers N=2,139	Carrying large cargo N=1,058	Running red lights N=648	Riding in a motor vehicle lane N=141	Mobile phone use N=116
Weather	Sunny	0.75 (0.54-1.05)	0.93 (0.57-1.53)	0.92 (0.54-1.55)	0.14 (0.01-2.19)	0.64 (0.09-4.39)	1.35 (0.05-37.20)
	Cloudy			reference			
Weekday	Yes	0.78 (0.56-1.08)	**0.46 (0.28-0.75)**	**0.53 (0.31-0.88)**	1.86 (0.12-28.19)	1.91 (0.32-11.50)	1.16 (0.04-36.12)
	No			reference			
Time of day	Morning	1.15 (0.90-1.48)	0.86 (0.59-1.25)	0.87 (0.59-1.30)	4.83 (0.58-40.31)	0.86 (0.19-3.99)	0.80 (0.06-10.56)
	Afternoon			reference			
Volume	Basic	1.15 (0.88-1.51)	1.04 (0.69-1.56)	0.97 (0.63-1.49)	0.11 (0.01-1.11)	0.35 (0.08-1.62)	2.55 (0.14-47.09)
	Low			reference			
Traffic control	Yes	**1.86 (1.36-2.54)**	1.33 (0.84-2.12)	**1.97 (1.20-3.21)**	**0.03 (0.00-0.39)**	**0.05 (0.01-0.40)**	1.26 (0.06-25.40)
	No			reference			
Gender	Male	**0.89 (0.81-0.97)**	**1.17 (1.05-1.29)**	1.14 (0.99-1.30)	**1.32 (1.11-1.57)**	1.13 (0.80-1.60)	1.75 (0.14-22.54)
	Female			reference			
Courier	Yes	0.71 (0.36-1.41)	not estimable	**11.06 (6.05-20.23)**	1.07 (0.37-3.12)	2.67 (0.62-11.53)	not estimable
	No			reference			
Registrations	Yes	**0.86 (0.78-0.93)**	**0.76 (0.68-0.84)**	**0.87 (0.76-0.99)**	1.03 (0.86-1.22)	**0.59 (0.40-0.88)**	0.98 (0.08-11.86)
	No			reference			
Pedals	Yes	0.92 (0.84-1.00)	**0.78 (0.71-0.87)**	**0.44 (0.38-0.50)**	0.91 (0.77-1.08)	0.95 (0.67-1.34)	0.99 (0.09-11.53)
	No			reference			

*Odds Ratios were adjusted for observational items in Table 1 (significant results are highlighted in bold).

For peer review only

DISCUSSION

Poor safety practice was commonplace including speeding, road rule violations, and little use of helmets and this did not vary between rural and urban areas. Male E-bikers seemed to bear more risks of speeding and road rule violations. Although couriers were 7 times more likely to wear a helmet when riding an E-bike, they were also 5 times more likely to violate road rules when entering an intersection compared with the other E-bikers. When riding E-bikes with pedals rather than those in scooter form, E-bikers had a lower likelihood of violating road rules and wearing a helmet. These identified safety gaps build on previous evidence [5-10] identifying the need to discourage unsafe practice and encourage safety gear use among E-bikers in China, particularly in the context of China recently joining global action to improve road safety in the next decade.[11]

Consistent with previous studies,[2, 8-10] this study confirmed a range of factors associated with observed E-biker behaviours and revealed the invariant nature of unsafe E-bike riding practice in general. We conducted a similar study during March 2012 in metropolitan Suzhou areas and found 27% of E-bikers violated at least one road rule and 41% used at least one type of safety gear.[10] The current study identified a somewhat higher prevalence of road rule violations (38%) and lower safety gear use (3%). This variation may be explained by the seasonality, e.g., the sharp drop in glove use (from 37% to 0.6%) and helmet use (from 9% to 2%). Zhang et al reported a similar decrease in helmet use among motorcycles in Guangxi during the hot and humid season.[14] The study findings also relate to the previous reports of increasing E-biker fatalities and injuries across mainland China,[4-7] which echoes the call for action to develop policies to improve E-bike safety in China.

Based on the study findings, measures aimed at improving e-bike safety are required. For example, the observed high prevalence of unsafe riding practices implies a need for policy change. Current road rules regulate E-bikes as pedal bicycles that should travel in non-motor vehicle lanes at a maximum speed of 15 km/h,[15] whereas the mandatory Standard for

For peer review only - http://bmjopen.bmj.com/site/about/guidelines.xhtml

E-bikes specifies a maximum speed of 20 Km/h and a maximum weight of 40 kilograms in addition to requiring a specified braking distance and pedal installment.[13] However, these specifications may not be widely enforced as most of the electric two-wheelers are not designed and produced in line with the national standards of non-motor vehicles.[16] With regard to those that do comply with the national standards of non-motor vehicles, producers, for marketing purposes, often install the so called “speed limiting devices” on their products. With the speed limiting devices, the maximum speed by which the electric two-wheelers could operate is 20 Km/h as required by the mandatory Standard for E-bikes; whereas, the speed limiting devices are designed and installed in a way that could be easily dismantled by customers themselves or sales persons. Without speed limiting devices, the speed of these electric two-wheelers could effortlessly go beyond 20 Km/h and maybe up to 40 Km/h [17].

Notably, the *Safety Specifications for Power Driven Vehicles Operating on Roads* defines a motorcycle as being power-driven with the maximum speed exceeding 50 Km/h and a moped with a maximum speed range from 20-50 Km/h.[18] This has the legal implication that any E-bikes (mostly in scooter form) that could travel faster than 20 Km/h r should be regulated as motor vehicles by the road rules. Obviously, such conflict between the mandatory Standard for E-bikes and road rules might create difficulties for legislative enforcement, including cities where motorcycles are banned.

In addition, the low use of helmets also implies a need for policy change given that the effectiveness of helmets in head injury prevention is well established for both bicyclists [19] and motorcyclists.[20] Regardless of the introduction of compulsory motorcycle helmet use producing a substantial increase in using of helmets among motorcyclists in China,[21] similar regulations were missing for bicyclists and E-bikers. Therefore, road rule revisions to encourage helmet use among E-bikers are urgently needed and should be incorporated into the broad road safety agenda.

For peer review only - http://bmjopen.bmj.com/site/about/guidelines.xhtml

To our best knowledge, no international E-bikers` riding practice studies were conducted before. Compared with other cross-sectional observational studies, this study is limited by lack of generalizability to other settings (different regions); possible bias due to unmeasurable confounding (influence of road infrastructure); and possible misclassification due to measurement errors (incorrect speed reading). Thus, care should be taken when interpreting the study findings. To minimize the likelihood of measurement errors, various small-scale pilot studies were conducted to determine the feasibility of the study and to validate the observational instruments. Moreover, this study established a strict quality control scheme and recruited experienced observers who had participated in previous studies using similar techniques.[10] Thus, misclassification may not bias the key findings to an important degree. Nevertheless, the study findings provide new evidence to complement previous findings as to diverse safety issues among E-bikers, and to reinforce the imperative to encourage safety gear use and discourage unsafe on-road practices.

CONCLUSION

E-bikes are becoming a dominant road transportation means for commuters in China, and they are increasingly used as a sustainable alternative to traditional transportation in other countries because of the low maintenance cost and low polluting mobility. The observed unsafe riding practices signal emerging road safety challenges in China and in similar settings elsewhere. Translating established safety practices such as helmet use and enforcing existing countermeasures such as speed limit devices may be used to improve safety practice among E-bikers. Strong political will is especially needed to leapfrog substantial losses associated with E-bike risk in China without sacrificing mobility needs.

For peer review only - http://bmjopen.bmj.com/site/about/guidelines.xhtml

ACKNOWLEDGEMENT

This project has funding support from Bloomberg Philanthropies as part of the Bloomberg Philanthropies Global Road Safety Programme in China. Additional funding was provided by Jiangsu Provincial Centre for Disease Control and Prevention (JKRC2011014). We appreciate special support from the WHO China Office, the Ministry of Health in China, Suzhou Bureau of Health, and Suzhou Bureau of Public Security. We also thank Ziyi Jin, Jianfeng Liu, Qi Zhang, Xianglin Liu, and Yan Lu for their assistance in field work. Wei Du is supported by an NHMRC fellowship. Yilan Liao is supported by the National Natural Science Foundation of China (No. 41101431）.

CONTRIBUTION

Jie Yang and Yihe Hu contributed equally to the study design, research implementation, literature review, data analysis, writing full first draft and contributed to subsequent drafts. All the other authors contributed to the conceptual development, data interpretation, critical revision of the first manuscript, and subsequent drafts.

COMPETING INTERESTS: There is no conflict of interest.

FUNDING: This work was funded by the Bloomberg Philanthropies Global Road Safety Programme, Jiangsu Provincial Centre for Disease Control and Prevention (JKRC2011014),National Natural Science Foundation of China (No. 41101431）

DATA SHARING STATEMENT: No additional data available.

For peer review only

For peer review only - http://bmjopen.bmj.com/site/about/guidelines.xhtml

REFERENCES

1 Weinert J, Ma C, Cherry C. The transition to electric bikes in China: history and key reasons for rapid growth. Transportation 2007;34: 301-318

2 2Yao L, Wu C. Traffic safety of e-bike riders in China: safety attitudes, risk perception and aberrant riding behaviors. Transportation Research Record: Journal of the Transportation Research Board & Transportation Research Board 91st Annual Meeting 2012: Paper 4599.

3 3Hurst D, Wheelock C. Electric two-wheel vehicles (electric bicycles, mopeds, scooters, and motorcycles): market analysis and forecasts. Boulder, Colorado: Pike Research, 2010.

4 Bureau of Traffic Management (2004-2010). China road traffic accidents statistics, 2004 to 2010. Beijing: Ministry of Public Security.

5 Du W, Yang J, Powis B, et al. Epidemiological profile of hospitalised injuries among electric bicycle riders admitted to a rural hospital in Suzhou: a cross sectional study. Injury Prevention 2013 Jun 1. [Epub ahead of print].

6 Duan L, Wu C, Deng X, et al. Road traffic injuries in China, 2006–2008. Chinese Journal of Public Health and Preventive Medicine 2010;21:10-13.

7 Feng Z, Raghuwanshi R, Xu Z, et al. Electric-bicycle-related injury: a rising traffic injury burden in China. Injury Prevention 2010;16: 417-419.

8 Weinert J, Ma C, Yang X, et al. Electric Two-Wheelers in China: Effect on Travel Behavior, Mode Shift, and User Safety Perceptions in a Medium-Sized City. Transportation Research Record: Journal of the Transportation Research Board 2007; 2038:62-68.

9 Wu C, Yao L, Zhang K. The red-light running behavior of electric bike riders and cyclists at urban intersections in China: An observational study. Accident Analysis & Prevention, Available online 2011 July 2.

10 Du W, Yang J, Powis B, et al. Understanding on-road practices of electric bike riders: an observational study in a developed city of China. Accident Analysis and Prevention 2013 Jun 25;59C:319-326. [Epub ahead of print]

11 World Health Organization. Global plan for the decade of action for road safety 2011-2020. Geneva: WHO, 2011. Available from:http://www.who.int/roadsafety/decade_of_action/plan/plan_english.pdf (accessed 10 Aug 2013)

12 http://www.bushnell.com/all-products/outdoor-technology/velocity-speed-gun. (accessed 1 Nov 2013)

13 Standardization Administration of the People’s Republic of China (1999). General technical standards of Electric bicycles (GB17761-1999). Chinese Standards Publisher, 1999.

14 Zhang J, Norton R, Tang K, et al. Motorcycle ownership and injury in China. Int. J. Injury Control and Safety Promotion 2004;11:159-163.

15 China National People’s Congress. Law of the People's Republic of China on road traffic safety. Beijing, 2004.

16 Tsinghua Law School. Aiming at Behavioral Changes: Improving Drink Driving and Speeding

For peer review only

For peer review only - http://bmjopen.bmj.com/site/about/guidelines.xhtml

Law in China Beijing: Tsinghua University, 2012 (Unpublished Report).

17 http://njrb.njnews.cn/html/2011-03/14/node_69.htm. (accessed 10 August 2013).

18 Standardization Administration of the People's Republic of China. Safety Specifications for Power Driven Vehicles Operating on Roads (GB7258-2004). Chinese Standards Publisher, 2004.

19 Thompson D, Rivara F, Thompson R. Helmets for preventing head and facial injuries in bicyclists. Cochrane Database of Systematic Reviews (published in Issue 4, 1999).

20 Liu B, Ivers R, Norton R,et al. Helmets for preventing injury in motorcycle riders. Cochrane Database of Systematic Reviews,(published in Issue 1, 2008).

21 Yu J, Ke L, Ivers R, et al. Prevalence rates of helmet use among motorcycle riders in a developed region in China. Accident Analysis and Prevention 2011;43:214-219.

For peer review only

For peer review only - http://bmjopen.bmj.com/site/about/guidelines.xhtml

Title

Unsafe riding practice among electric bikers in Suzhou, China: an observational study

Corresponding Author

Dr. Ming Wu

Tel/Fax: +86 (25) 8375 9411

Email: wuming@jscdc.cn

Address: Department of Non-communicated Chronic Disease Prevention and Control, Jiangsu Provincial Center for Disease Prevention and Control, Nanjing 210009, China

Authors

Jie Yang, MSc[1*], Yihe Hu, MBBS[2*], Wei Du, PhD[3,4], Brent Powis, PhD[5], Joan Ozanne-Smith, MD[6], Yilan Liao, PhD[7], Ning Li, PhD[3], Ming Wu, PhD[1]

1. Department of Chronic Disease Control, Jiangsu Provincial Centre for Disease Control and Prevention, China
2. Department of Chronic Disease Control, Suzhou Municipal Centre for Disease Control and Prevention, Suzhou, China
3. Institute of Population Research, Peking University, China
4. Neuroscience Research Australia, University of New South Wales, Australia
5. School of Postgraduate Studies and Research, International Medical University, Malaysia
6. Department of Forensic Medicine, Monash University, Australia
7. Institute of Geographical Sciences and Nature Resources Research, Chinese Academy of Sciences, China

* These two authors contributed equally

Key Words: Electric Bike, Cross Sectional Study, Behavior, Risk Factor Research, Driver.

Word count: 2,705 words

For peer review only - http://bmjopen.bmj.com/site/about/guidelines.xhtml

Abstract

Background: Increasing Electric bike (E-bike) related fatalities have been increasing rapidly in China and such injuries may be partly attributable to unsafe riding practice.

Objectives: To describe potentially unsafe riding behaviors among E-bikers and to investigate factors influencing these practices in China.

Methods: In September 2012, a cross-sectional observation study including a speed measurement component was conducted in Wuzhong (an urban District) and Zhangjiagang (a rural District) of Suzhou, Jiangsu Province, China. Hand held radar speed meters were used to read traveling speeds of E-bikes and a pro-forma observation checklist was used to collect data on road riding practice. Mixed-effect logistic regressions were used to calculate adjusted Odds Ratios (ORs) and 95% Confidence Intervals (CIs) for the association between speeding, road rule violations and helmet use and their influencing factors.

Results: Among 800 E-bikes with a speed reading, 70.9% exceeded the designed speed limit of 20 km/h. Among a further 20,647 E-bikers observed, 38.3% did not comply with the road rules when entering intersections; and only 2.2% wore helmets. No regional variation was identified between urban and rural areas. Male was associated with more speeding and road rule violations; whereas riding a pedal-equipped E-bike was associated with less road rule violations and less helmet use.

Conclusion: Unsafe riding practices such as speeding, road rule violations, and lack of helmet use were commonplace among E-bikers, especially among males. The study findings indicate that measures aimed at improving e-bike safety are required in China.

For peer review only - http://bmjopen.bmj.com/site/about/guidelines.xhtml

Article Summary

Article Focus:

- E-biker safety is an emerging public health challenge in China. This study focuses on the unsafe on-road riding behaviours among Chinese E-bikers and factors influencing these practices.

Key Message:

- Direct roadside observation techniques were applied to describe safety practices of E-bikers and hand held radar meters were used to estimate their actual travelling speed.
- The variation of on-road riding behaviours between rural and urban areas was evaluated.
- Factors influencing observed riding behaviours were further investigated.

Strengths and Limitations of the Study:

- In this study, we evaluated how fast E-bikers ride on roads and the possible regional variation of riding behaviours. Furthermore, we investigated factors influencing observed riding behaviours.
- Study findings could provide new evidence to enhance the understanding of on-road riding behaviours among Chinese E-bikers, and to reinforce the imperative to encourage safety gear use and discourage unsafe on-road practices.
- Findings might be limited by lack of generalizability to other settings, possible bias due to unmeasurable confounding, and possible misclassification due to measurement errors.

For peer review only

INTRODUCTION

In China, the last few years have witnessed the rapid growth of E-bikes (either with pedals or in scooter form) due to increasing mobility demand when public transportation systems are crowded and inconveniently routed.[1] Growing wealth among Chinese also increases affordability of E-bike purchase, normally priced at approximately USD 300. In China, more than 120 million E-bikes were registered by 2011 [2] and globally an estimated 466 million E-bikes are expected to hit the road by 2016. [3]

In China, bicycle use is shifting to E-bike use. Unfortunately, an associated unwanted shift was observed as E-bike related fatalities increased almost 7 times over time from 589 in 2004 to 4,029 in 2010 across the nation, whereas bicycle related fatalities decreased 3 times approximately from 13,655 to 4616 during the same period. [4] Moreover, E-bikers hospitalized for injuries accounted for 57% of serious non-fatal road traffic injuries and 50% of the direct hospitalization cost for all road crash casualties in a rural hospital in Suzhou.[5] Thus, E-biker safety is an emerging public health challenge in China .[5-7]

Whilst unsafe riding practices have been reported among E-bikers using a self-reported survey,[2,8] synchronized video camera recording techniques,[9] and direct roadside observations,[10] no studies have reported on how fast E-bikers ride on roads allowing for possible regional variation, such as rural/urban disparities, commonly observed for other road safety issues. To enhance the understanding of on-road riding behaviors among Chinese E-bikers including evaluation of rural/urban variation, we used direct roadside observation techniques to describe their safety practices and hand held radar meters to estimate their actual traveling speed. We further investigated factors influencing these observed behaviors.

METHODS

We applied a cross-sectional observation research which comprised two components for this study, i.e., observations *with* or *without* speed measurement, which were conducted separately in Suzhou, one of the intervention pilot cities in China for the Bloomberg

For peer review only - http://bmjopen.bmj.com/site/about/guidelines.xhtml

Philanthropies Global Road Safety Programme (A multinational programme which take effort to reduce death and serious injury on the roads in ten low- and middle-income countries over five years extending from 2010 to 2014). [11] Suzhou has the sixth highest gross domestic product (GDP) per capita on the Chinese mainland, a resident population of 10 million and at least 2 million E-bikes. [10] The study protocol was approved by the Ethics Committee of Jiangsu Provincial Centre for Disease Control and Prevention.

For peer review only

Field implementation

In Suzhou, two administrative districts, i.e., Wuzhong (urban district) and Zhangjiagang (rural district) were selected to conduct both study components. Wuzhong is located in the south of Suzhou metropolitan regions with a resident population of 606,231 in 2012, comprising 49.0% males and 51.0% females; whereas Zhangjiagang is located to the north of Suzhou metropolitan regions with a resident population of 909,038 in 2012, comprising 49.2% males and 50.8% females. To select observation sites, a grid was placed over standard maps of Wuzhong and Zhangjiagang, random digits were generated for each grid box for selection and each valid grid box contained at least one intersection having traffic lights. For each randomly selected site, an alternate site was also selected randomly from the grid as a backup. A pilot study was carried out to validate the field feasibility such as having low volumes of E-bikes for speed measurement; at least two-way motor vehicle lanes, pedestrian crossings, and bicycle lanes; enough distance between observation sites so the same E-bikers were unlikely to be observed twice; less likely to interrupt observed behaviors and least likely to increase the crash risk for observers. A total of eight sites (i.e, two from each district for each study component) were randomly selected.

For the study component with speed measurement, observers concealed themselves at approximately 50 meters from the corner of the intersection, used handheld radar speed meters (Bushnell Velocity 10-1911CM with measurement range of 16-320km/h) [12] to record the speed meter reading, and collected information on on-road riding behaviors among oncoming E-bikers with valid speed meter reading. For the study component without speed

For peer review only - http://bmjopen.bmj.com/site/about/guidelines.xhtml

measurement, the observations were conducted at intersection corners. Considering traffic characteristics may vary every day, we randomly selected 4 days in a week including one weekend day (September 11th, 12th, 13th, and 15th) for the study component with speed measurement and selected another 7-day period (September 17th-23th) for the study component without speed measurement. The time of day (7.00am–6.59pm) for site observations was set at 2-hour intervals as observational periods and randomly assigned to sites.

Four experienced observers were recruited, who had participated in previous roadside E-bike observation studies in other districts in Suzhou.[10] Prior to field implementation, the site observers were trained in specifications of different behaviors, identification of different types of protective items, techniques to observe multiple behaviors especially when an E-bike was moving, and data quality control. The site observers formed two groups, i.e., the urban group and rural group without rotation. Each group remained in the same district and changed their observational periods and sites every day. Roadside pilot observations and regular on-site audits were conducted to ensure the safety guidelines and accurate and appropriate implementation of the data collection process.

The observers worked in pairs, to observe oncoming E-bikes in ascending distance order. Data items were collected on a pro-forma checklist including type of E-bike (with bike pedals or in scooter form), registration status, rider's gender, couriers or not (In China, many couriers are required to wear uniforms when working and companies provide them uniforms with own logos; although uniforms are in different styles, it's easy to differentiate couriers from normal E-bikers), carrying passengers, carrying oversized cargo (estimated >60 x 40 x 20cm^3, the size of normal airlines carry-on luggage), riding in a motor vehicle lane, running red lights, riding in the opposite direction (i.e., facing oncoming traffic), using mobile phone, using helmet, wearing leather gloves, and wearing other motorcycle protective clothing. Weather, day of week, time of day, average E-bike traffic volume per minute, presence of a traffic controller (traffic policeman or traffic police assistant directing vehicular and pedestrian traffic) were recorded on separate data collection forms.[Table 1] Regarding the speed measurement

For peer review only - http://bmjopen.bmj.com/site/about/guidelines.xhtml

component, for every tenth E-bike, the second observer recorded the radar speed reading during the speed measurement observations.

Outcome of interest

1) Speeding was defined as binary, i.e., yes (travel speed >20 km/h) or no (travel speed ≤20km/h), because E-bikes are manufactured to a mandatory Standard [13] with designed maximum travel speed of 20 km/h;
2) Violation was defined as binary, i.e., yes (at least one of the following road rule violations was observed: carrying passengers, carrying oversized cargo, riding in a motor vehicle lane, running red lights, riding in the opposite direction or using mobile phone), or no;
3) Helmet use was defined as binary, i.e., yes (wearing a motorcycle helmet) or no.

Table 1. Observational item categorization

Observational items	Categorization
weather	sunny, cloudy, or rainy
day of week	weekday or weekend
time of day	morning or afternoon
average E-bike traffic volume per minute	basic (≤10 E-bikes), low (11-15 E-bikes), medium (16-20 E-bikes), or high (>20 E-bikes)
presence of a traffic controller (traffic policeman or traffic police assistant directing vehicular and pedestrian traffic)	yes or no
type of E-bike	equipped with pedals or otherwise in scooter form
E-bike registration status	registered with registration plate displayed or otherwise unregistered;
E-bikers' gender	male or female
E-bikers' occupation	couriers or not
carrying passengers	yes or no
carrying oversized cargo (>60 x 40 x 20cm^3)	yes or no
riding in a motor vehicle lane	yes or no
running red lights	yes or no
riding in the opposite direction (i.e., facing oncoming traffic)	yes or no
mobile phone use	yes or no
helmet use	yes or no
wearing leather gloves	yes or no
wearing other motorcycle protective clothing	yes or no

Statistical analysis

Completed observational records were reviewed; and data were entered with double entry. All

For peer review only - http://bmjopen.bmj.com/site/about/guidelines.xhtml

data analyses were conducted using SAS version 9.2 (SAS Institute, 2002). The inter-observer reliability was assessed using Kappa statistics and agreement reached at least 85% for each pair of observers. Frequencies and proportions of speed reading and observed on-road riding behaviors were calculated where appropriate. We used mixed logistic regression allowing for random selection of observational sites to estimate Odds Ratio (OR) and associated 95% Confidence Interval (CI) for different study outcomes adjusted for observational items. [Table 1] Further mixed-effect logistic regression analyses of individual road rule violations (i.e., carrying passengers, carrying oversized cargo, riding in a motor vehicle lane, running red lights, riding in the opposite direction or using mobile phone) were stratified by different regions, i.e., Wuzhong or Zhangjiagang. We defined P-values less than 0.05 as statistically significant.

RESULTS

There were a total of 27 observational periods (i.e., 14 in Wuzhong and 13 in Zhangjiagang) for direct observational data collection on 20,647 E-bikes, and 16 periods (i.e., 8 for each district) for speed measurement on 800 E-bikes. The average number of E-bikes per observational period was 729 (range: 103 to 1317) and 803 (range: 552 to 1046) for Wuzhong and Zhangjiagang, respectively.

Table 2 Distribution of observational items among E-bike study populations

		Wuzhong (Urban district)		Zhangjiagang (Rural district)	
		with speed measure	without speed measure	with speed measure	without speed measure
Sample size	(n)	400	10202	400	10445
Traffic mix	E-bikes (%)	44.6	46.5	28.2	34.5
	Pedestrians (%)	7.4	9.1	3.0	10.6
	Bicycles (%)	3.5	3.2	9.6	4.7
	Cars (%)	36.2	35.1	57.1	47.6
	Heavy vehicles （%）	8.3	6.2	2.2	2.7
E-bike volume per minute (basic as ≤10 E-bikes, low as 11-15 E-bikes, medium as16-20 E-bikes, or high as >20 E-bikes)	Basic (%)	25.0	40.9	50.0	40.2
	Low (%)	12.5	17.0	37.5	59.8
	Medium (%)	37.5	10.4	12.5	0.0
	High (%)	25.0	31.7	0.0	0.0

For peer review only

Weather	Sunny (%)	75.0	82.8	100.0	69.8
	Cloudy (%)	0.0	9.4	0.0	30.2
	Rainy (%)	25.0	7.8	0.0	0.0
Day of week	Weekday (%)	75.0	75.7	75.0	70.5
Time of day	Morning (%)	37.5	45.7	50.0	39.5
Traffic controller	Yes (%)	Not applicable	0.0	Not applicable	25.4
Occupation	Courier (%)	1.8	0.8	1.3	0.5
Gender	Males (%)	69.0	59.8	58.5	51.7
Registration	Yes (%)	94.0	96.3	35.3	36.3
Pedals	Yes (%)	28.0	37.1	53.3	54.3
Carrying passengers	Yes (%)	24.3	21.3	11.0	20.5
Carrying large cargo	Yes (%)	11.5	6.9	15.8	10.1
Riding in a motor vehicle lane	Yes (%)	13.5	3.1	2.3	1.4
Riding opposite direction	Yes (%)	3.0	5.7	25.0	30.8
Mobile phone use	Yes (%)	0.8	0.7	2.0	1.1
Helmet use	Yes (%)	3.3	2.1	5.0	2.2
Gloves	Yes (%)	0.8	0.4	1.8	0.8
Running red lights	Yes (%)	Not applicable	16.5	Not applicable	6.2

Table 2 describes the observation results and shows that E-bikes were the dominant transportation means in Wuzhong district. Despite similarities across some observational items such as low helmet use (i.e., 2.1% in Wuzhong vs 2.2% in Zhangjiagang) and commonplace carrying of passengers (21.3% vs 20.5%), there were differences in riding violations especially for riding licensed E-bikes (96.3% vs 36.3%) [Table 2].

Approximately 83.3% (n=333) and 58.5% (n=234) E-bikers were observed traveling at a speed greater than 20 km/h; approximately 41.3% (n=4,211) and 35.4% (n=3,700) violating at least one of the listed road rules; and 2.5% (n=251) and 3.1% (n=319) using any safety gear in Wuzhong and Zhangjiagang, respectively.

No statistically significant evidence indicates the existence of regional variation in terms of elevated odds of speeding, general road rule violations, or lack of helmet use. Compared with female E-bikers, males showed greater ORs of speeding (OR=2.12, 95%CI=1.50-3.01) and violation (OR=1.35, 95%CI=1.27-1.44). Reduced ORs of road rule violations (OR=0.66, 95%CI=0.62-0.70) and wearing a helmet (OR=0.39, 95%CI=0.32-0.49) were found to be associated with riding pedal-equipped E-bikes compared with those in scooter form; whereas the highest elevated ORs of Helmet use (OR=7.21,95%CI=4.01-12.98) and road rule

For peer review only - http://bmjopen.bmj.com/site/about/guidelines.xhtml

violations (OR=5.34, 95%CI=3.58-7.99) were observed among couriers compared with other E-bikers [Table 3].

Table 3. Adjusted Odds Ratios (95% Confidence Intervals) for speeding, road rule violations, and helmet use among E-bikers *

		Speeding N=800	Violations N=20,647	Helmet use N=20,647
Region	Urban	1.14 (0.66-1.99)	1.01 (0.95-1.07)	0.98 (0.84-1.14)
	Rural		reference	
Weather	Sunny	0.29 (0.02-3.58)	0.89 (0.40-1.98)	0.58 (0.19-1. 80)
	Cloudy	--	0.76 (0.31-1.88)	0.55 (0.16-1.98)
	Rainy		reference	
Weekday	Yes	1.73 (0.92-3.25)	0.86 (0.53-1.42)	1.43 (0.72-2.81)
	No		reference	
Time of day	Morning	0.73 (0.47-1.15)	1.07 (0.69-1.65)	1.01 (0.56-1.82)
	Afternoon		reference	
Volume	Basic	0.27 (0.02-3.24)	1.53 (0.77-3.02)	0.86 (0.35-2.14)
	Low	0.50 (0.04-6.11)	1.29 (0.62-2.67)	0.90 (0.34-2.37)
	Medium	0.16 (0.01-2.06)	1.13 (0.36-3.60)	0.66 (0.14-3.02)
	High		reference	
Traffic control	Yes	--	0.76 (0.41-1.38)	1.57 (0.70-3.51)
	No		reference	
Gender	Male	**2.12 (1.50-3.01)**	**1.35 (1.27-1.44)**	0.66 (0.54-0.80)
	Female		reference	
Courier	Yes	0.75 (0.20-2.82)	**5.34 (3.58-7.99)**	**7.21 (4.01-12.98)**
	No		reference	
Registration	Yes	0.96 (0.63-1.44)	**0.82 (0.75-0.88)**	1.18 (0.92-1.52)
	No		reference	
Pedals	Yes	0.79 (0.56-1.12)	**0.66 (0.62-0.70)**	**0.39 (0.32-0.49)**
	No		reference	

* The adjusting variables were observational items showed in Table1.
Note: Significant results are highlighted in bold

The results of regional stratification demonstrate that risk factor profile may vary across regions. For example of riding opposite direction, couriers were associated with significantly elevated OR (2.03, 95%CI=1.03-4.00) in Wuzhong but marginally reduced OR (0.71, 95%CI=0.36-1.41) in Zhangjiagang; whereas males were associated with marginally elevated (1.09, 95%CI=0.91-1.31) in Wuzhong but significantly reduced OR (0.89, 95%CI=0.81-0.97) in Zhangjiagang [Tables 4 and 5].

For peer review only - http://bmjopen.bmj.com/site/about/guidelines.xhtml

Table 4. Adjusted Odds Ratios (95% Confidence Intervals) for individual road rule violations among E-bikers observed in Wuzhong (Urban districtu)*

		Carrying passengers N=2,169	Running red lights N=1,682	Carrying large cargo N=700	Riding opposite direction N=579	Riding in a motor vehicle lane N=315	Mobile phone use N=72
Weather	Sunny	2.51 (0.64-9.86)	0.41 (0.06-2.58)	1.16 (0.62-2.18)	1.54 (0.49-4.84)	1.03 (0.07-15.00)	0.80 (0.16-4.05)
	Cloudy	2.01 (0.32-12.74)	0.78 (0.07-9.51)	1.28 (0.56-2.93)	1.43 (0.31-6.62)	7.72 (0.21-288.20)	3.17 (0.34-29.69)
	Rainy			reference			
Weekday	Yes	0.55 (0.16-1.92)	1.21 (0.22-6.62)	1.15 (0.64-2.06)	0.83 (0.29-2.36)	0.45 (0.04-5.31)	0.98 (0.14-6.69)
	No			reference			
Time of day	Morning	0.71 (0.22-2.33)	2.35 (0.47-11.70)	1.22 (0.70-2.10)	0.90 (0.34-2.42)	0.86 (0.08-8.97)	1.60 (0.32-8.00)
	Afternoon			reference			
Volume	Basic	0.53 (0.15-1.92)	9.21 (1.63-51.92)	**3.59 (2.01-6.41)**	0.90 (0.31-2.59)	**47.54 (3.69-612.34)**	5.15 (1.13-23.42)
	Low	0.65 (0.11-4.01)	4.95 (0.43-57.61)	**2.65 (1.12-6.25)**	0.86 (0.19-3.98)	3.86 (0.11-133.67)	3.51 (0.32-37.89)
	Medium	0.43 (0.07-2.76)	8.41 (0.68-104.11)	**1.88 (0.81-4.36)**	0.83 (0.18-3.89)	7.59 (0.20-296.13)	4.67 (0.43-50.91)
	High			reference			
Gender	Male	**1.26 (1.13-1.40)**	**1.32 (1.17-1.49)**	**1.73 (1.43-2.10)**	1.09 (0.91-1.31)	**2.14 (1.62-2.84)**	**2.38 (1.29-4.40)**
	Female			reference			
Courier	Yes	**0.11 (0.03-0.43)**	0.82 (0.44-1.55)	**53.36 31.34-90.86)**	**2.03 (1.03-4.00)**	1.14 (0.34-3.76)	**5.26 (1.79-15.46)**
	No			reference			
Registrations	Yes	1.17 (0.89-1.54)	0.99 (0.75-1.32)	**0.69 (0.48-0.99)**	1.07 (0.68-1.66)	0.74 (0.45-1.23)	3.16 (0.43-22.94)
	No			reference			
Pedals	Yes	**0.69 (0.62-0.77)**	0.89 (0.79-1.01)	**0.62 (0.51-0.75)**	0.99 (0.83-1.20)	**0.57 (0.43-0.77)**	0.82 (0.47-1.42)
	No			reference			

* Odds Ratios were adjusted for observational items in Table 1 (significant results are highlighted in bold).

For peer review only

Table 5. Adjusted Odds Ratios (95% Confidence Intervals) for individual road rule violations among E-bikers observed in Zhangjiagang (Rural district)*

		Riding opposite direction N=3,220	Carrying passengers N=2,139	Carrying large cargo N=1,058	Running red lights N=648	Riding in a motor vehicle lane N=141	Mobile phone use N=116
Weather	Sunny	0.75 (0.54-1.05)	0.93 (0.57-1.53)	0.92 (0.54-1.55)	0.14 (0.01-2.19)	0.64 (0.09-4.39)	1.35 (0.05-37.20)
	Cloudy			reference			
Weekday	Yes	0.78 (0.56-1.08)	**0.46 (0.28-0.75)**	**0.53 (0.31-0.88)**	1.86 (0.12-28.19)	1.91 (0.32-11.50)	1.16 (0.04-36.12)
	No			reference			
Time of day	Morning	1.15 (0.90-1.48)	0.86 (0.59-1.25)	0.87 (0.59-1.30)	4.83 (0.58-40.31)	0.86 (0.19-3.99)	0.80 (0.06-10.56)
	Afternoon			reference			
Volume	Basic	1.15 (0.88-1.51)	1.04 (0.69-1.56)	0.97 (0.63-1.49)	0.11 (0.01-1.11)	0.35 (0.08-1.62)	2.55 (0.14-47.09)
	Low			reference			
Traffic control	Yes	**1.86 (1.36-2.54)**	1.33 (0.84-2.12)	**1.97 (1.20-3.21)**	**0.03 (0.00-0.39)**	**0.05 (0.01-0.40)**	1.26 (0.06-25.40)
	No			reference			
Gender	Male	**0.89 (0.81-0.97)**	**1.17 (1.05-1.29)**	1.14 (0.99-1.30)	**1.32 (1.11-1.57)**	1.13 (0.80-1.60)	1.75 (0.14-22.54)
	Female			reference			
Courier	Yes	0.71 (0.36-1.41)	not estimable	**11.06 (6.05-20.23)**	1.07 (0.37-3.12)	2.67 (0.62-11.53)	not estimable
	No			reference			
Registrations	Yes	**0.86 (0.78-0.93)**	**0.76 (0.68-0.84)**	**0.87 (0.76-0.99)**	1.03 (0.86-1.22)	**0.59 (0.40-0.88)**	0.98 (0.08-11.86)
	No			reference			
Pedals	Yes	0.92 (0.84-1.00)	**0.78 (0.71-0.87)**	**0.44 (0.38-0.50)**	0.91 (0.77-1.08)	0.95 (0.67-1.34)	0.99 (0.09-11.53)
	No			reference			

*Odds Ratios were adjusted for observational items in Table 1 (significant results are highlighted in bold).

For peer review only - http://bmjopen.bmj.com/site/about/guidelines.xhtml

DISCUSSION

Poor safety practice was commonplace including speeding, road rule violations, and little use of helmets and this did not vary between rural and urban areas. Male E-bikers seemed to bear more risks of speeding and road rule violations. Although couriers were 7 times more likely to wear a helmet when riding an E-bike, they were also 5 times more likely to violate road rules when entering an intersection compared with the other E-bikers. When riding E-bikes with pedals rather than those in scooter form, E-bikers had a lower likelihood of violating road rules and wearing a helmet. These identified safety gaps build on previous evidence [5-10] identifying the need to discourage unsafe practice and encourage safety gear use among E-bikers in China, particularly in the context of China recently joining global action to improve road safety in the next decade.[11]

Consistent with previous studies,[2, 8-10] this study confirmed a range of factors associated with observed E-biker behaviours and revealed the invariant nature of unsafe E-bike riding practice in general. We conducted a similar study during March 2012 in metropolitan Suzhou areas and found 27% of E-bikers violated at least one road rule and 41% used at least one type of safety gear.[10] The current study identified a somewhat higher prevalence of road rule violations (38%) and lower safety gear use (3%). This variation may be explained by the seasonality, e.g., the sharp drop in glove use (from 37% to 0.6%) and helmet use (from 9% to 2%). Zhang et al reported a similar decrease in helmet use among motorcycles in Guangxi during the hot and humid season.[14] The study findings also relate to the previous reports of increasing E-biker fatalities and injuries across mainland China,[4-7] which echoes the call for action to develop policies to improve E-bike safety in China.

Based on the study findings, measures aimed at improving e-bike safety are required. For example, the observed high prevalence of unsafe riding practices implies a need for policy change. Current road rules regulate E-bikes as pedal bicycles that should travel in non-motor vehicle lanes at a maximum speed of 15 km/h,[15] whereas the mandatory Standard for

For peer review only - http://bmjopen.bmj.com/site/about/guidelines.xhtml

E-bikes specifies a maximum speed of 20 Km/h and a maximum weight of 40 kilograms in addition to requiring a specified braking distance and pedal installment.[13] However, these specifications may not be widely enforced as most of the electric two-wheelers are not designed and produced in line with the national standards of non-motor vehicles.[16] With regard to those that do comply with the national standards of non-motor vehicles, producers, for marketing purposes, often install the so called “speed limiting devices” on their products. With the speed limiting devices, the maximum speed by which the electric two-wheelers could operate is 20 Km/h as required by the mandatory Standard for E-bikes; whereas, the speed limiting devices are designed and installed in a way that could be easily dismantled by customers themselves or sales persons. Without speed limiting devices, the speed of these electric two-wheelers could effortlessly go beyond 20 Km/h and maybe up to 40 Km/h [17].

Notably, the *Safety Specifications for Power Driven Vehicles Operating on Roads* defines a motorcycle as being power-driven with the maximum speed exceeding 50 Km/h and a moped with a maximum speed range from 20-50 Km/h.[18] This has the legal implication that any E-bikes (mostly in scooter form) that could travel faster than 20 Km/h r should be regulated as motor vehicles by the road rules. Obviously, such conflict between the mandatory Standard for E-bikes and road rules might create difficulties for legislative enforcement, including cities where motorcycles are banned.

In addition, the low use of helmets also implies a need for policy change given that the effectiveness of helmets in head injury prevention is well established for both bicyclists [19] and motorcyclists.[20] Regardless of the introduction of compulsory motorcycle helmet use producing a substantial increase in using of helmets among motorcyclists in China,[21] similar regulations were missing for bicyclists and E-bikers. Therefore, road rule revisions to encourage helmet use among E-bikers are urgently needed and should be incorporated into the broad road safety agenda.

For peer review only - http://bmjopen.bmj.com/site/about/guidelines.xhtml

To our best knowledge, no international E-bikers` riding practice studies were conducted before. Compared with other cross-sectional observational studies, this study is limited by lack of generalizability to other settings (different regions); possible bias due to unmeasurable confounding (influence of road infrastructure); and possible misclassification due to measurement errors (incorrect speed reading). Thus, care should be taken when interpreting the study findings. To minimize the likelihood of measurement errors, various small-scale pilot studies were conducted to determine the feasibility of the study and to validate the observational instruments. Moreover, this study established a strict quality control scheme and recruited experienced observers who had participated in previous studies using similar techniques.[10] Thus, misclassification may not bias the key findings to an important degree. Nevertheless, the study findings provide new evidence to complement previous findings as to diverse safety issues among E-bikers, and to reinforce the imperative to encourage safety gear use and discourage unsafe on-road practices.

CONCLUSION

E-bikes are becoming a dominant road transportation means for commuters in China, and they are increasingly used as a sustainable alternative to traditional transportation in other countries because of the low maintenance cost and low polluting mobility. The observed unsafe riding practices signal emerging road safety challenges in China and in similar settings elsewhere. Translating established safety practices such as helmet use and enforcing existing countermeasures such as speed limit devices may be used to improve safety practice among E-bikers. Strong political will is especially needed to leapfrog substantial losses associated with E-bike risk in China without sacrificing mobility needs.

ACKNOWLEDGEMENT

This project has funding support from Bloomberg Philanthropies as part of the Bloomberg Philanthropies Global Road Safety Programme in China. Additional funding was provided by Jiangsu Provincial Centre for Disease Control and Prevention (JKRC2011014). We

For peer review only - http://bmjopen.bmj.com/site/about/guidelines.xhtml

For peer review only

appreciate special support from the WHO China Office, the Ministry of Health in China, Suzhou Bureau of Health, and Suzhou Bureau of Public Security. We also thank Ziyi Jin, Jianfeng Liu, Qi Zhang, Xianglin Liu, and Yan Lu for their assistance in field work. Wei Du is supported by an NHMRC fellowship. Yilan Liao is supported by the National Natural Science Foundation of China (No. 41101431）.

CONTRIBUTION

Jie Yang and Yihe Hu contributed equally to the study design, research implementation, literature review, data analysis, writing full first draft and contributed to subsequent drafts. All the other authors contributed to the conceptual development, data interpretation, critical revision of the first manuscript, and subsequent drafts.

COMPETING INTERESTS: There is no conflict of interest.

REFERENCES

1 Weinert J, Ma C, Cherry C. The transition to electric bikes in China: history and key reasons for rapid growth. Transportation 2007;34: 301-318

2 2Yao L, Wu C. Traffic safety of e-bike riders in China: safety attitudes, risk perception and aberrant riding behaviors. Transportation Research Record: Journal of the Transportation Research Board & Transportation Research Board 91st Annual Meeting 2012: Paper 4599.

3 3Hurst D, Wheelock C. Electric two-wheel vehicles (electric bicycles, mopeds, scooters, and motorcycles): market analysis and forecasts. Boulder, Colorado: Pike Research, 2010.

4 Bureau of Traffic Management (2004-2010). China road traffic accidents statistics, 2004 to 2010. Beijing: Ministry of Public Security.

5 Du W, Yang J, Powis B, et al. Epidemiological profile of hospitalised injuries among electric bicycle riders admitted to a rural hospital in Suzhou: a cross sectional study. Injury Prevention 2013 Jun 1. [Epub ahead of print].

6 Duan L, Wu C, Deng X, et al. Road traffic injuries in China, 2006–2008. Chinese Journal of Public Health and Preventive Medicine 2010;21:10-13.

7 Feng Z, Raghuwanshi R, Xu Z, et al. Electric-bicycle-related injury: a rising traffic injury burden in China. Injury Prevention 2010;16: 417-419.

8 Weinert J, Ma C, Yang X, et al. Electric Two-Wheelers in China: Effect on Travel Behavior,

For peer review only - http://bmjopen.bmj.com/site/about/guidelines.xhtml

For peer review only

Mode Shift, and User Safety Perceptions in a Medium-Sized City. Transportation Research Record: Journal of the Transportation Research Board 2007; 2038:62-68.

9 Wu C, Yao L, Zhang K. The red-light running behavior of electric bike riders and cyclists at urban intersections in China: An observational study. Accident Analysis & Prevention, Available online 2011 July 2.

10 Du W, Yang J, Powis B, et al. Understanding on-road practices of electric bike riders: an observational study in a developed city of China. Accident Analysis and Prevention 2013 Jun 25;59C:319-326. [Epub ahead of print]

11 World Health Organization. Global plan for the decade of action for road safety 2011-2020. Geneva: WHO, 2011. Available from:http://www.who.int/roadsafety/decade_of_action/plan/plan_english.pdf (accessed 10 Aug 2013)

12 http://www.bushnell.com/all-products/outdoor-technology/velocity-speed-gun. (accessed 1 Nov 2013)

13 Standardization Administration of the People's Republic of China (1999). General technical standards of Electric bicycles (GB17761-1999). Chinese Standards Publisher, 1999.

14 Zhang J, Norton R, Tang K, et al. Motorcycle ownership and injury in China. Int. J. Injury Control and Safety Promotion 2004;11:159-163.

15 China National People's Congress. Law of the People's Republic of China on road traffic safety. Beijing, 2004.

16 Tsinghua Law School. Aiming at Behavioral Changes: Improving Drink Driving and Speeding Law in China Beijing: Tsinghua University, 2012 (Unpublished Report).

17 http://njrb.njnews.cn/html/2011-03/14/node_69.htm. (accessed 10 August 2013).

18 Standardization Administration of the People's Republic of China. Safety Specifications for Power Driven Vehicles Operating on Roads (GB7258-2004). Chinese Standards Publisher, 2004.

19 Thompson D, Rivara F, Thompson R. Helmets for preventing head and facial injuries in bicyclists. Cochrane Database of Systematic Reviews (published in Issue 4, 1999).

20 Liu B, Ivers R, Norton R,et al. Helmets for preventing injury in motorcycle riders. Cochrane Database of Systematic Reviews,(published in Issue 1, 2008).

21 Yu J, Ke L, Ivers R, Du W, Senserrick T. Prevalence rates of helmet use among motorcycle riders in a developed region in China. Accident Analysis and Prevention 2011;43:214-219.

For peer review only

For peer review only - http://bmjopen.bmj.com/site/about/guidelines.xhtml

STROBE 2007 (v4) Statement—Checklist of items that should be included in reports of *cross-sectional studies*

**Section/Topic**	**Item #**	**Recommendation**	**Reported on page #**
**Title and abstract**	1	(*a*) Indicate the study's design with a commonly used term in the title or the abstract	2
		(*b*) Provide in the abstract an informative and balanced summary of what was done and what was found	2
**Introduction**			
Background/rationale	2	Explain the scientific background and rationale for the investigation being reported	4
Objectives	3	State specific objectives, including any prespecified hypotheses	4
**Methods**			
Study design	4	Present key elements of study design early in the paper	5
Setting	5	Describe the setting, locations, and relevant dates, including periods of recruitment, exposure, follow-up, and data collection	5
Participants	6	(*a*) Give the eligibility criteria, and the sources and methods of selection of participants	5,6
Variables	7	Clearly define all outcomes, exposures, predictors, potential confounders, and effect modifiers. Give diagnostic criteria, if applicable	7
Data sources/ measurement	8*	For each variable of interest, give sources of data and details of methods of assessment (measurement). Describe comparability of assessment methods if there is more than one group	6,7
Bias	9	Describe any efforts to address potential sources of bias	*13*
Study size	10	Explain how the study size was arrived at	7
Quantitative variables	11	Explain how quantitative variables were handled in the analyses. If applicable, describe which groupings were chosen and why	6,7
Statistical methods	12	(*a*) Describe all statistical methods, including those used to control for confounding	8
		(*b*) Describe any methods used to examine subgroups and interactions	8
		(*c*) Explain how missing data were addressed	Not applicable
		(*d*) If applicable, describe analytical methods taking account of sampling strategy	Not applicable
		(*e*) Describe any sensitivity analyses	Not applicable
**Results**			

For peer review only

Participants	13*	(a) Report numbers of individuals at each stage of study—eg numbers potentially eligible, examined for eligibility, confirmed eligible, included in the study, completing follow-up, and analysed	8
		(b) Give reasons for non-participation at each stage	Not applicable
		(c) Consider use of a flow diagram	Not applicable
Descriptive data	14*	(a) Give characteristics of study participants (eg demographic, clinical, social) and information on exposures and potential confounders	8,9
		(b) Indicate number of participants with missing data for each variable of interest	-
Outcome data	15*	Report numbers of outcome events or summary measures	8,9
Main results	16	(*a*) Give unadjusted estimates and, if applicable, confounder-adjusted estimates and their precision (eg, 95% confidence interval). Make clear which confounders were adjusted for and why they were included	10
		(*b*) Report category boundaries when continuous variables were categorized	Yes
		(*c*) If relevant, consider translating estimates of relative risk into absolute risk for a meaningful time period	Not applicable
Other analyses	17	Report other analyses done—eg analyses of subgroups and interactions, and sensitivity analyses	Not applicable
**Discussion**			
Key results	18	Summarise key results with reference to study objectives	11
Limitations	19	Discuss limitations of the study, taking into account sources of potential bias or imprecision. Discuss both direction and magnitude of any potential bias	13
Interpretation	20	Give a cautious overall interpretation of results considering objectives, limitations, multiplicity of analyses, results from similar studies, and other relevant evidence	13
Generalisability	21	Discuss the generalisability (external validity) of the study results	14
**Other information**			
Funding	22	Give the source of funding and the role of the funders for the present study and, if applicable, for the original study on which the present article is based	14

*Give information separately for cases and controls in case-control studies and, if applicable, for exposed and unexposed groups in cohort and cross-sectional studies.

Note: An Explanation and Elaboration article discusses each checklist item and gives methodological background and published examples of transparent reporting. The STROBE checklist is best used in conjunction with this article (freely available on the Web sites of PLoS Medicine at http://www.plosmedicine.org/, Annals of Internal Medicine at http://www.annals.org/, and Epidemiology at http://www.epidem.com/). Information on the STROBE Initiative is available at www.strobe-statement.org.

For peer review only
